# Metabolic effects of leptin receptor knockdown or reconstitution in adipose tissues

**DOI:** 10.1038/s41598-019-39498-3

**Published:** 2019-03-01

**Authors:** Sandra Pereira, Shannon M. O’Dwyer, Travis D. Webber, Robert K. Baker, Victor So, Cara E. Ellis, Ji Soo Yoon, Majid Mojibian, Maria M. Glavas, Subashini Karunakaran, Susanne M. Clee, Scott D. Covey, Timothy J. Kieffer

**Affiliations:** 1 Department of Cellular and Physiological Sciences, University of British Columbia, Vancouver, BC Canada; 2 Department of Biochemistry and Molecular Biology, University of British Columbia, Vancouver, BC Canada; 3 Department of Surgery, University of British Columbia, Vancouver, BC Canada

## Abstract

The relative contribution of peripheral and central leptin signalling to the regulation of metabolism and the mechanisms through which leptin affects glucose homeostasis have not been fully elucidated. We generated complementary lines of mice with either leptin receptor (Lepr) knockdown or reconstitution in adipose tissues using Cre-lox methodology. Lepr knockdown mice were modestly lighter and had lower plasma insulin concentrations following an oral glucose challenge compared to controls, despite similar insulin sensitivity. We rendered male mice diabetic using streptozotocin (STZ) and found that upon prolonged leptin therapy, Lepr knockdown mice had an accelerated decrease in blood glucose compared to controls that was associated with higher plasma concentrations of leptin and leptin receptor. Mice with transcriptional blockade of Lepr (*Lepr*^*loxTB/loxTB*^) were obese and hyperglycemic and reconstitution of Lepr in adipose tissues of *Lepr*^*loxTB/loxTB*^ mice resulted in males reaching a higher maximal body weight. Although mice with adipose tissue Lepr reconstitution had lower blood glucose levels at several ages, their plasma insulin concentrations during an oral glucose test were elevated. Thus, attenuation or restoration of Lepr in adipocytes alters the plasma insulin profile following glucose ingestion, modifies the glucose-lowering effect of prolonged leptin therapy in insulin-deficient diabetes, and may modulate weight gain.

## Introduction

Multiple studies have reported that leptin action in the central nervous system (CNS) is critical for its regulation of food intake, thermogenesis, glucose homeostasis, and insulin sensitivity^1–8^. The contribution of peripheral leptin signalling to the regulation of body weight and glucose metabolism has not been fully elucidated. From the single leptin receptor (*Lepr*) gene, multiple isoforms of Lepr exist, and it is believed that the long isoform (Leprb) is responsible for the majority of leptin’s actions^9,10^. Using a tamoxifen-inducible model, where *Leprb* expression was reduced to different extents in peripheral tissues, but was not affected in the CNS, Guo *et al*.^11^ observed no alterations in metabolism, including insulin sensitivity, except for hyperleptinemia, which was higher in females. The hyperleptinemia resulted from increased leptin secretion from adipose tissue, possibly due to interference in a negative feedback loop, but leptin action was apparently not affected because plasma levels of free leptin were not changed. Mice with liver-specific Lepr knockdown on standard chow have improved insulin sensitivity and glucose tolerance, but when on high fat diet, only the latter is ameliorated^12^. Using antisense RNA expressed under the control of the phosphoenolpyruvate carboxykinase (PEPCK) promoter, Huan *et al*.^13^ downregulated expression of leptin receptor isoforms in mouse white adipose tissue (WAT). This resulted in obesity, insulin resistance, glucose intolerance, and ectopic fat accumulation. However, it has been suggested that the antisense mRNA approach to reduce gene expression, as used by Huan *et al*.^13^, may itself lead to obesity in rodents^14^. Wang *et al*.^15^ reported that overexpression of Leprb in adipose tissue using an aP2 promoter inhibited the elevation in body weight caused by high fat diet, but these alterations may not have been adipose tissue-specific because subsequently, studies with *aP2-Cre* mice also found Cre-mediated recombination in non-adipose tissues^16^. In the current study, our objective was to clarify the role of adipose tissue leptin signalling in metabolism using two complementary approaches based on Cre-lox methodology: generating mice lacking Leprb in adipose tissues and mice that express Leprb only in adipose tissues.

## Methods

### Study design

Mice expressing Cre under the control of the Adiponectin promoter (*AdipoqCre*) were obtained from The Jackson Laboratory (Bar Harbor, ME, USA, stock# 010803). *AdipoqCre* mice produce recombination selectively in white and brown adipose tissues^1,17^. *AdipoqCre* mice were mated with: 1) mice with floxed exon 17 of the leptin receptor gene (*Lepr*^*flox/flox*^)^5,12,18^ to generate mice that have diminished expression of Leprb due to excision of exon 17 in adipose tissues (AdipoqKO colony) and 2) mice containing a transcriptional blockade of the leptin receptor gene (*Lepr*^*loxTB/loxTB*^, The Jackson Laboratory, stock#018989)^8^ to generate mice that express leptin receptors only in adipose tissues (ATLeprEXP colony). All *AdipoqCre*^+^ mice studied were heterozygous for Cre. Littermate controls were used for all studies. *AdipoqCre*, *Lepr*^*flox/flox*^, and *Lepr*^*loxTB/loxTB*^ mice were on a mixed C57BL/6 and FVB background; contribution of 129 was minor. Ear notch samples were obtained to determine genotypes and genotyping is described in Supplementary Information. To assess Cre activity by immunofluorescence, *AdipoqCreLepr*^*flox/flox*^ mice were mated with *mTmG* mice^19,20^, which were on a mixed C57BL/6J and 129 background, and *AdipoqCre*^+^*Lepr*^*flox/flox*^*ROSA26*^*mTmG/mTmG*^ as well as *AdipoqCre*^−^*Lepr*^*flox/flox*^*ROSA26*^*mTmG/mTmG*^ mice were studied. Breeders were fed Harlan diet 2919 and maintenance diet was standard chow (Harlan #2918). For high fat diet (HFD) and low fat diet (LFD) studies, mice were placed on HFD (60 kcal%, Cat# D12492i, Research Diets) or LFD (10 kcal%, Cat# D12450Bi, Research Diets) at 9 weeks of age. Mice were housed in a 12 h:12 h light-dark cycle, food and water were available *ad libitum*, and mice were euthanized following a 4 h fast unless stated otherwise. All procedures were approved by the University of British Columbia Animal Care Committee and followed the guidelines of the Canadian Council on Animal Care.

### Streptozotocin (STZ) and leptin therapy studies

Two studies were performed to investigate the effects of leptin therapy in STZ-induced diabetes: (1) In the prolonged leptin therapy study, at 10–12 weeks of age, male *AdipoqCre*^+^*Lepr*^*flox/flox*^ mice and their *AdipoqCre*^−^*Lepr*^*flox/flox*^ male littermates were given 180 mg per kg body weight of STZ intraperitoneally (i.p.) (Sigma-Aldrich)^21^ on Day −8, while controls were not injected. Diabetes in STZ-injected mice was defined as blood glucose concentrations ≥18.0 mM following a 4 h morning fast on Days −3 and −1. On Day 0, diabetic STZ-mice were implanted subcutaneously with mini-osmotic pumps (DURECT Corporation, Cupertino, CA, USA) containing either recombinant murine leptin (Peprotech, Rocky Hill, NJ, USA) released at a dose of 20 μg/day or vehicle, as previously described^21^, for 8 days. Mice that did not receive STZ underwent sham surgery on Day 0, whereby a skin incision was made, subcutaneous tunneling was done, and finally, the opening was sutured. (2) In the acute leptin therapy study, STZ (180 mg per kg body weight, i.p.) was administered to aged-matched (13–36 weeks old) male *AdipoqCre*^+^*Lepr*^*flox/flox*^ and *AdipoqCre*^−^*Lepr*^*flox/flox*^ mice on Day -8. Criteria for diabetes was the same as for the prolonged leptin therapy study. On Day 0, following a 4 h morning fast, mice were injected i.p. with recombinant murine leptin (Peprotech) at a dose of 3 mg per kg body weight, as utilized by Burnett *et al*.^22^. Plasma was obtained from saphenous vein blood collected immediately before injecting leptin (0 h), as well as at 0.25 h, 0.5 h, 1 h, 2 h, 4 h, and 6 h after injecting leptin. Half-life of leptin was calculated using a one phase decay curve^22^ (GraphPad Prism 7), with plasma leptin concentrations from 0.5 h (peak of average plasma leptin) to 6 h following leptin injection.

### Immunofluorescence analysis and hematoxylin and eosin (H&E) staining

Methodology for these studies are found in Supplemental Material.

### Plasma assays

Body weight and blood glucose measurements were obtained following a 4 h morning fast. Blood glucose was measured in samples obtained from saphenous vein blood using a One Touch Ultra Glucometer (Life Scan, Burnaby, Canada). When blood glucose concentration exceeded the limit of detection of the glucometer (>33.3 mM), it was assigned to equal 33.3 mM. Plasma was obtained from saphenous vein blood or from cardiac blood collected following a 4 h morning fast unless stated otherwise. Plasma insulin was measured using the mouse ultrasensitive insulin ELISA from ALPCO Diagnostics (Salem, NH, USA) and plasma leptin was measured using the mouse leptin ELISA from Crystal Chem (Downers Grove, IL, USA). Plasma insulin concentrations of mice from the ATLeprEXP colony at 10 weeks of age and during glucose tolerance tests were measured using the Stellux Chemi Rodent Insulin ELISA (Alpco). The assay for plasma free fatty acids (FFAs) (Wako Chemicals, Richmond, VA, USA) and the assay for glycerol and triglycerides (TGs) (Sigma-Aldrich) were performed as previously stated^23^. For analyses of plasma obtained from cardiac blood in the ATLeprEXP colony, a custom-made multiplex assay for mouse leptin, insulin, and resistin (Milliplex, EMD Millipore, Billerica, MA, USA) was used according to the manufacturer’s instructions; undiluted samples from all mice were assessed for insulin and resistin, while samples were diluted 1:8 for leptin assay. The concentration of leptin receptor in plasma was determined with a mouse leptin receptor ELISA from R&D Systems (Minneapolis, MN, USA; Catalogue #DY008 and #DY497) and by following the manufacturer’s instructions.

### *In vivo* metabolic tests

Mice were fasted for 4 h, starting in the morning, before performing i.p. or oral glucose tolerance tests (IPGTTs and OGTTs, respectively) or i.p. insulin tolerance tests (ITTs, 0.65 or 0.75U human synthetic insulin (Novolin ge Toronto, Novo Nordisk, Mississauga, Canada) per kg body weight)^12,24^. The higher insulin dose was used in older mice due to age-associated insulin resistance. For mice in the AdipoqKO colony, which contains mice with Lepr knockdown, the dose for IPGTTs and OGTTs was 1.5 g glucose per kg body weight. For mice in the ATLeprEXP colony, which contains mice with Lepr reconstitution, the dose for IPGTTs and OGTTs was 1 g glucose per kg body weight. Doses for IPGTTs, OGTTs, and ITTs were normalized for body weight and consequently, in studies where body weight differed between groups, the absolute doses differed between groups. Area under the curve (AUC) was calculated using GraphPad Prism 7; baseline was 0 mM for glucose and 0 ng/ml for insulin. For fasting-refeeding experiments, mice were fasted starting at 5:30–6:30 pm and blood was obtained at 0 h (immediately before start of fast), 4, 12, and 16 h of fasting. Food was then given to the mice and blood samples were obtained 1 h and 2 h following the refeeding. Blood obtained from the saphenous vein was used for all *in vivo* metabolic tests.

### Metabolic cages and DEXA analysis

In order to assess food intake, activity, energy expenditure, and substrate utilization, mice were placed in PhenoMaster metabolic cages (TSE Systems) at 23 °C, as previously described^21,25^. Dual energy x-ray absorbance (DEXA) measurements (Lunar PIXImus 2.0 Densitometer, Inside Outside Sales, Madison, WI, USA) were obtained in the non-fasting state. For mice from the AdipoqKO colony, DEXA analysis was carried out following metabolic cage studies, on the same day.

### Western blot analysis and hepatic glycogen content

Female *AdipoqCre*^+^*Lepr*^*flox/flox*^ and *AdipoqCre*^−^*Lepr*^*flox/flox*^ mice were fasted overnight for 16 h, anesthetized with isoflurane, and injected with either 200 µl of 5U per kg body weight insulin (Novolin ge Toronto) or vehicle via the portal vein^26,27^. Vehicle consisted of 0.1% fatty acid-free bovine serum albumin (BSA; Equitech-Bio Inc, Kerrville, TX, USA), prepared by dissolving BSA in 0.9% NaCl. One minute following injection, livers were freeze-clamped and stored at −80 °C. Approximately 50 mg of each liver sample was homogenized with the following buffer: 1X RIPA lysis buffer (EMD Millipore, Burlington, MA, USA), Complete mini EDTA-free protease inhibitor cocktail (Roche, Basel, Switzerland), 1 µg/ml pepstatin A (Sigma), 20 mM sodium fluoride (Sigma), 2 mM sodium pyrophosphate (Sigma), and 25 mM β-glycerophosphate (Sigma). Samples were rocked at 4 °C for 40 min, centrifuged at 12,000 *g* and 4 °C for 10 min, and lastly, the supernatant of each sample was collected and frozen at −80 °C. The Bradford method was used to measure protein concentration (Bio-Rad, Hercules, CA, USA). Samples were mixed in a 1:1 ratio with 2X Laemmli buffer containing 10% β-mercaptoethanol and boiled for 5 min before electrophoretic separation. After protein transfer to polyvinylidene fluoride membranes, membranes were blocked (Odyssey blocking buffer, Li-Cor, Lincoln, NE, USA) and incubated overnight at 4 °C with phospho-Akt (Ser473) antibody (Cell Signaling Technology, Danvers, MA, USA; Catalogue #4060, 1:2000 dilution). Tris-buffered saline with Tween (TBST) was used to wash the membranes and the ensuing incubation with secondary antibody (IRDye 800CW goat anti-rabbit IgG, Li-Cor; Catalogue #925-32211, 1:10000 dilution) lasted 1 h at room temperature. Membranes were washed before imaging (Odyssey Classic Infrared Imaging System, Li-Cor). Afterwards, membranes were stripped (NewBlot PVDF stripping buffer, Li-Cor), blocked, and incubated overnight at 4 °C with an Akt antibody that detects total Akt protein (Cell Signaling Technology; Catalogue #2920, 1:2000 dilution). The rest of the steps taken for imaging have been described above, except the secondary antibody was IRDye 680RD goat anti-mouse IgG (Li-Cor; Catalogue #925-68070, 1:10000 dilution). ImageJ (National Institutes of Health, Bethesda, MD, USA) was used to quantify bands; quantification of bands for insulin- or vehicle-injected mice were normalized to bands of *AdipoqCre*^−^*Lepr*^*flox/flox*^ mice that received the same type of injection. Liver samples from these mice were also used to determine hepatic glycogen content according to the manufacturer’s instructions (BioVision, Milpitas, CA, USA).

### Endpoint PCR and isolation of adipocytes from WAT

*AdipoqCre*^+^*Lepr*^*flox/flox*^ and *AdipoqCre*^−^*Lepr*^*flox/flox*^ mice were euthanized and tissues were immediately collected, placed in liquid nitrogen, and stored at −80 °C. Subcutaneous WAT (scWAT) was obtained from the inguinal region and brown adipose tissue (BAT) was obtained from the interscapular region. Subsequently, DNA was extracted from WAT, BAT, adipocyte fractions of WAT, hypothalamus, liver, skeletal muscle (gastrocnemius), heart, and pancreas^28^ and endpoint PCR was carried out using primers previously described to determine *Lepr* recombination^12^. Adipocyte fractions were isolated from WAT based on Ruan *et al*.^29^. Briefly, freshly obtained scWAT and perigonadal WAT (pgWAT) samples were minced in collagenase solution, which consisted of 2 mg/mL collagenase Type I (Worthington, Lakewood, NJ, USA) dissolved in a working solution (pH = 7.4) of Krebs-Ringer-Phosphate-HEPES buffer, 200 μM adenosine (Sigma, St. Louis, MO, USA), and 2.5% bovine serum albumin (Millipore, Billerica, MA, USA). Afterwards, samples were incubated at 37 °C until most of the adipose tissue pieces were not visible, which took approximately 40 min. Digestion was stopped by washing three times with working solution. The floating cells (adipocytes) were allowed to rise to the top for 1–2 minutes and adipocytes together with <1 mL of working solution were collected. For assessment of recombination in gastrointestinal tissues by endpoint PCR and qPCR, female and male *AdipoqCre*^+^*Lepr*^*flox/flox*^ and *AdipoqCre*^−^*Lepr*^*flox/flox*^ mice were fasted overnight for 16 h and injected with 0.1% BSA intraportally, as described in the *Western blot analysis* section above. Intestinal mucosa was collected because Leprb and adiponectin are expressed in the epithelium of small and large intestines^30–33^. The lumen of the small and large intestine was flushed with phosphate buffered saline, each section of the intestine was cut longitudinally, and then the mucosa was scraped using a glass microscope slide. Collected tissues were immediately placed in liquid nitrogen, stored at −80 °C, and subsequently DNA was extracted^28^.

### qPCR

We developed an assay to assess the extent of excision of *Lepr*^*flox/flox*^, that is, *Lepr* knockdown in DNA isolated^28^ from pgWAT, BAT, and gastrointestinal tissues (*AdipoqCre*^+^*Lepr*^*flox/flox*^ vs. *AdipoqCre*^−^*Lepr*^*flox/flox*^ mice). The intron 17 probe is an internal reference gene and the intron 16 probe anneals to the intact floxed Lepr gene, but not the excised Lepr gene. The LeprInt17-FAM probe is 5′ 56-FAM/TAGGGCGGA/ZEN/TGAACCAGCAAATGT/3IABkFQ and the LeprInt16-HEX probe is 5′/5HEX/AGGAACTTCG/ZEN/GAATAGGAACTTCGAATTCCTCGAGATC/3IABkFQ. Primers used are as follows: LeprInt17-F, 5′CCTTTCCAGATAATGCCTGATAGA3′; LeprInt17-R, 5′GCACCACACTTAGCTCCAATA3′; LeprInt16-F, 5′GATCTCACACATACCAGATCC3′; LeprInt16-R, 5′ATTTGATTCCACAAAGTGTTCC3′. A master mix was created using SsoAdvanced universal probes supermix (Bio-Rad). The Pfaffl equation, which takes into account the different efficiencies of the two primer sets, was used to calculate the extent of recombination. Another assay was created to determine the extent of excision of *Lepr*^*loxTB/loxTB*^, that is, *Lepr* reconstitution in DNA isolated^28^ from pgWAT, scWAT, and BAT of *AdipoqCre*^+^*Lepr*^*loxTB/loxTB*^ mice. Two primer sets were used. The first primer set quantifies *Lepr*^*loxTB*^ in a sample and is as follows: 5′GGAAGATCTGGACTCTAGATAAGTAATGA3′ and 5′CAACAATTGCATTCATTTTATGTTTCAGG3′. The second primer set quantifies the recombined *Lepr*^*loxTB*^ allele (*Lepr*^*loxTBΔ*^) and is as follows: 5′TCATGGCAATGGACCAATGA3′ and 5′TCAAGACCATCTATCAAATCAGACA3′. A master mix was generated using SsoFast evagreen supermix (Bio-Rad). Since efficiency was 97% for the first set of primers and 100% for the second set of primers, calculation of the extent of recombination assumed an efficiency of 100% for both primer sets. The calculation was as follows: % recombination = [FD/(1 + FD)], where FD = fold difference and FD = 2^[Cq(*LeprloxTB*)-Cq(*LeprloxTBΔ*)]^.

### RT-qPCR

RNA was extracted from pgWAT and BAT using the RNeasy lipid tissue mini kit (Qiagen, Hilden, Germany) and cDNA was subsequently generated (iScript cDNA synthesis kit, Bio-Rad). SsoFast evagreen supermix (Bio-Rad) was used for each master mix and the sequences of the primers are as follows: *Fasn* (fatty acid synthetase), 5′ACAGATGATGACAGGAGATGGAAG3′ and 5′TCATAGCTGACTTCCAACAGCA3′; *Pnpla2* (adipose triglyceride lipase), 5′CCAACGCCACTCACATCTAC3′ and 5′GATGGTCTTCACCAGGTTGAAG3′; *Slc2a4* (GLUT4), 5′CAAGATGCCGTCGGGTTTC3′ and 5′GTTGCATTGTAGCTCTGTTCAATCA3′; *Ucp1* (uncoupling protein 1), 5′GGCCCTTGTAAACAACAAAATAC3′ and 5′GGCAACAAGAGCTGACAGTAAAT3′; *Ppia* (peptidylpropyl isomerase A; reference transcript), 5′AGCTCTGAGCACTGGAGAGA3′ and 5′GCCAGGACCTGTATGCTTTA3′. The efficiency of each primer set was obtained using cDNA from wild-type controls and the Pfaffl equation was used to calculate expression relative to wild-type controls.

### *Ex vivo* lipolysis assay, *ex vivo* leptin secretion, and hepatic lipid extraction

For the *ex vivo* lipolysis assay, *AdipoqCre*^+^*Lepr*^*flox/flox*^ and *AdipoqCre*^−^*Lepr*^*flox/flox*^ mice, aged 20–28 weeks, were euthanized after a ~4–8 h fast. The *ex vivo* lipolysis assay protocol was performed as described by Sakaguchi *et al*.^34^, except that ~20–35 mg of each adipose tissue depot were used and the dose of isoprenaline (Sigma) was 0.1 μM. The FFA and glycerol assays used for plasma samples were also used to determine FFA and glycerol concentrations in the collected media. FFA and glycerol concentrations in the media were divided by the tissue weight. Leptin in these media was assayed with a mouse leptin ELISA (Crystal Chem) and the results were divided by tissue weight. We have previously described the hepatic lipid extraction protocol in Huynh *et al*.^12^; lipid content was divided by weight of liver tissue sample.

### Statistical analyses

Data are presented as mean ± SEM. Statistical analyses consisted of unpaired t-test (or Mann-Whitney *U* test), Pearson correlation (or Spearman correlation), one-way ANOVA with Tukey’s *post-hoc* test, or repeated measures two-way ANOVA with *post-hoc* testing, as appropriate. Comparisons between two genotypes were performed using unpaired t-tests, unless distributions of a given parameter deviated substantially from normality in one or both genotypes, in which case the Mann-Whitney *U* test was used (SPSS 25). Pearson correlation, or Spearman correlation if large deviations from normality existed in one or both genotypes, were also performed. When comparing a parameter among more than 2 groups at a given timepoint, one-way ANOVA with Tukey’s *post-hoc* test was used. Repeated measures two-way ANOVA, with group as the between-subject variable and time as the within-subject variable, was performed with our research question being whether groups differed over time for each parameter (GraphPad Prism 7). The *post-hoc* analysis of repeated measures two-way ANOVA was done when the main effect of group and/or an interaction was statistically significant and on each such occasion, groups were compared at each timepoint with either Bonferroni (2 groups) or Tukey (more than 2 groups) adjustment for multiple comparisons. Where repeated measures two-way ANOVA could not be done due to missing data points, an alternative analysis was done using R software and is described in Supplementary Information. ANCOVA, with body weight as co-variate, was performed for heat and food intake (SPSS 25). Significance was achieved when p < 0.05.

## Results

### *Lepr* recombination

**Figure 1:**
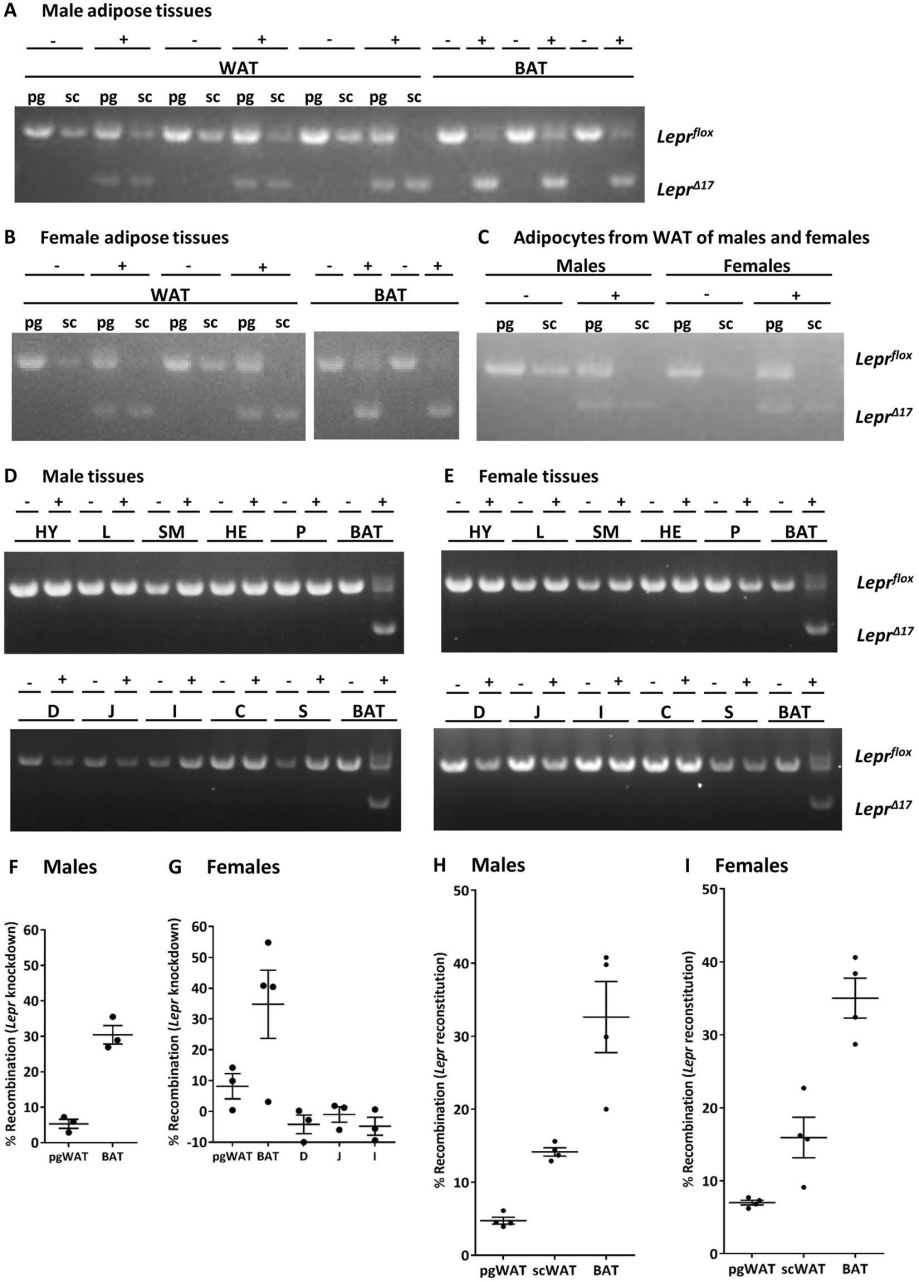
(**A**–**E**) Endpoint PCR indicating the location on the gel of *Lepr*^*flox*^ (1369 bp) and the allele resulting from Cre-induced excision, *Lepr*^*Δ17*^ (952 bp), in white adipose tissue (WAT), brown adipose tissue (BAT), adipocytes isolated from WAT, and non-adipose tissues in male and female *AdipoqCre*^+^*Lepr*^*flox/flox*^ (+) and *AdipoqCre*^−^*Lepr*^*flox/flox*^ (−) mice. Results in (**A**–**E**) are from 6 separate gels and the full-length blots/gels are presented in Supplementary Fig. S1. In A, results are from one single gel; in B, results are from the second gel that has been cropped and the white space indicates cropping; in C, results are from the third gel; in D and E, results are from the fourth gel that has been cropped and the white space indicates cropping, and from the fifth and sixth gels. (**F**–**I**) Extent of *Lepr* recombination in *AdipoqCre*^+^*Lepr*^*flox/flox*^ and *AdipoqCre*^+^*Lepr*^*loxTB/loxTB*^ mice determined by qPCR. pg, perigonadal; sc, subcutaneous; HY, hypothalamus; L, liver; SM, skeletal muscle (gastrocnemius); HE, heart; P, pancreas; D, duodenum mucosa; J, jejunum mucosa; I, ileum mucosa; C, colon mucosa; S, stomach.

*Lepr* knockdown was observed in WAT, adipocytes isolated from WAT, and BAT of *AdipoqCre*^+^*Lepr*^*flox/flox*^ male and female mice, but not in non-adipose tissues (Fig. 1A–G). Although it has been reported that adiponectin and Lepr are expressed in the intestines^30–33^, we did not find recombination in the gastrointestinal tract (Fig. 1D,E,G and Supplementary Fig. S2) and the results obtained with qPCR, which were within assay error, agree with those obtained with endpoint PCR for the same samples (Fig. 1G and Supplementary Fig. S2). *Lepr* reconstitution was found in WAT and BAT of *AdipoqCre*^+^*Lepr*^*loxTB/loxTB*^ male and female mice (Fig. 1H,I) and the extent of recombination was similar to that observed in *AdipoqCre*^+^*Lepr*^*flox/flox*^ mice. The greatest amount of recombination was observed in BAT. We also mated mice from the AdipoqKO colony with *mTmG* mice to assess GFP immunofluorescence as a marker of Cre activity in WAT. Although GFP immunofluorescence was not uniform, GFP immunofluorescence confirmed Cre activity in adipocytes of pgWAT and scWAT (representative images in Supplementary Fig. S3). Adipocyte size differences were not obviously discernible between genotypes in pgWAT, scWAT, and BAT (representative images in Supplementary Fig. S3).

### Mice with adipose tissue-specific *Lepr* knockdown

**Figure 2:**
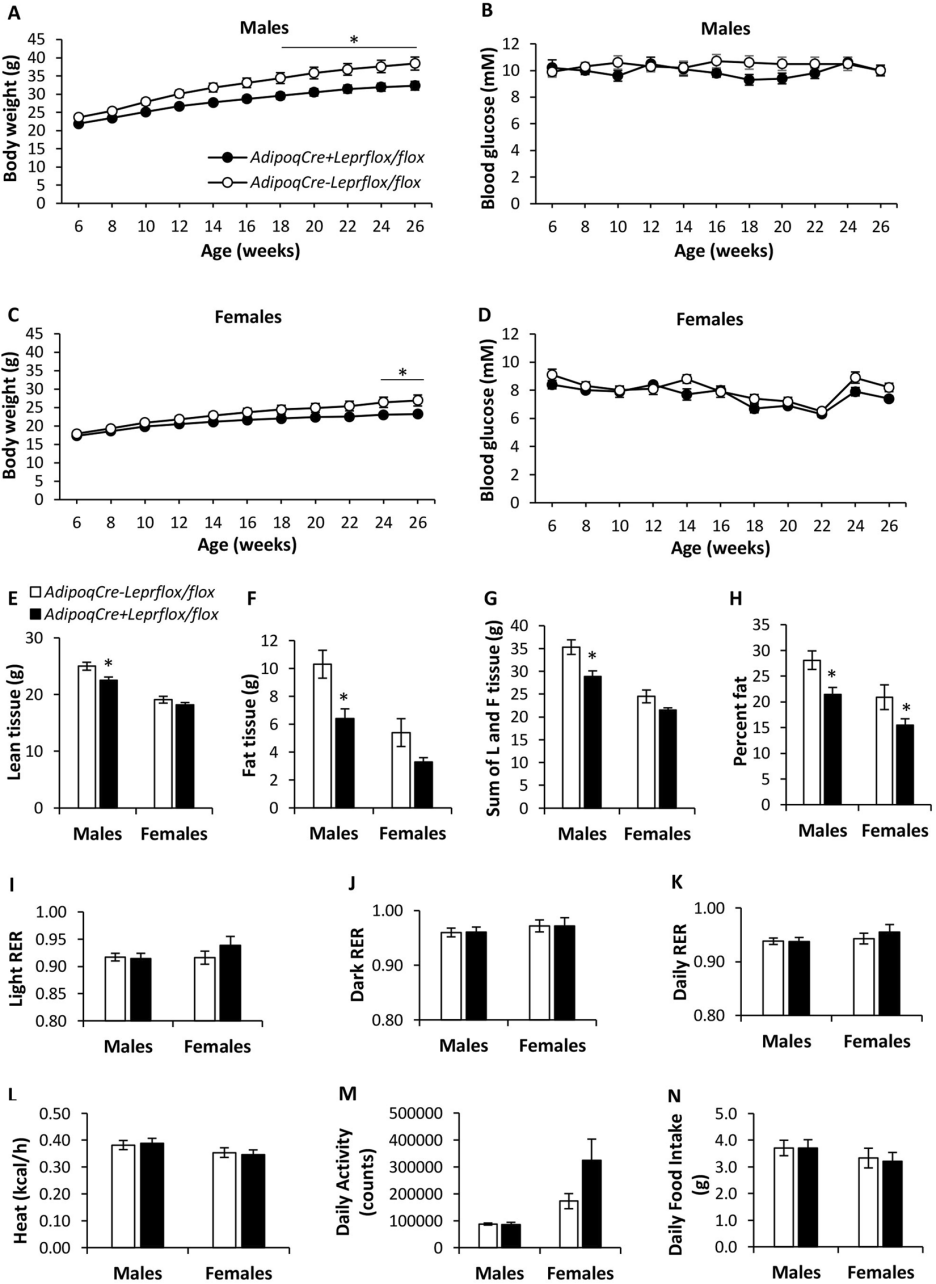
Body weight and blood glucose of male (**A**,**B**) and female (**C**,**D**) *AdipoqCre*^−^*Lepr*^*flox/flox*^ and *AdipoqCre*^+^*Lepr*^*flox/flox*^ mice after a 4 h fast. Body composition (**E**–**H**) and metabolic cage parameters (**I**–**N**) of *AdipoqCre*^−^*Lepr*^*flox/flox*^ and *AdipoqCre*^+^*Lepr*^*flox/flox*^ mice; male mice were 28–30 weeks old and female mice were 32 weeks old. For males, n = 15 for *AdipoqCre*^−^*Lepr*^*flox/flox*^ and n = 13 for *AdipoqCre*^+^*Lepr*^*flox/flox*^ mice. For females, n = 11 for *AdipoqCre*^−^*Lepr*^*flox/flox*^ and n = 13 for *AdipoqCre*^+^*Lepr*^*flox/flox*^ mice. In (**A**–**D**), repeated measures two-way ANOVA with Bonferroni *post-hoc* test was performed. For heat and food intake, an ANCOVA, using the non-fasting body weight obtained on the day metabolic cage experiments were started as co-variate, was performed and estimated marginal means ± SEM are presented. For other parameters, an unpaired t-test was performed, except for Females in (**E**,**F**,**H**), where the Mann-Whitney *U* test was used. *p < 0.05 vs. *AdipoqCre*^−^*Lepr*^*flox/flox*^ of same sex. In (**D**), there is a main effect of time (p < 0.05). L, lean; F, fat.

Body weight was lower in *AdipoqCre*^+^*Lepr*^*flox/flox*^ (knockdown) vs. *AdipoqCre*^−^*Lepr*^*flox/flox*^ (Flox control) male mice starting at 18 weeks of age and lower in female knockdown mice vs. female Flox controls at 24 and 26 weeks of age (p < 0.05; Fig. 2A,C). Blood glucose concentrations did not differ between genotypes at different ages for each sex (Fig. 2B,D). These mice subsequently underwent DEXA and metabolic cage assessments. In male knockdown mice, lean tissue mass, fat tissue mass, sum of lean and fat tissue, and percent fat were significantly lower compared to controls (p < 0.05; Fig. 2E–H). In females, differences in these parameters only reached statistical significance for percent fat, which was lower in the knockdown mice (p < 0.05). Respiratory exchange ratio (RER), heat, activity, and food intake were similar between genotypes, for each sex (Fig. 2I–N).

**Figure 3:**
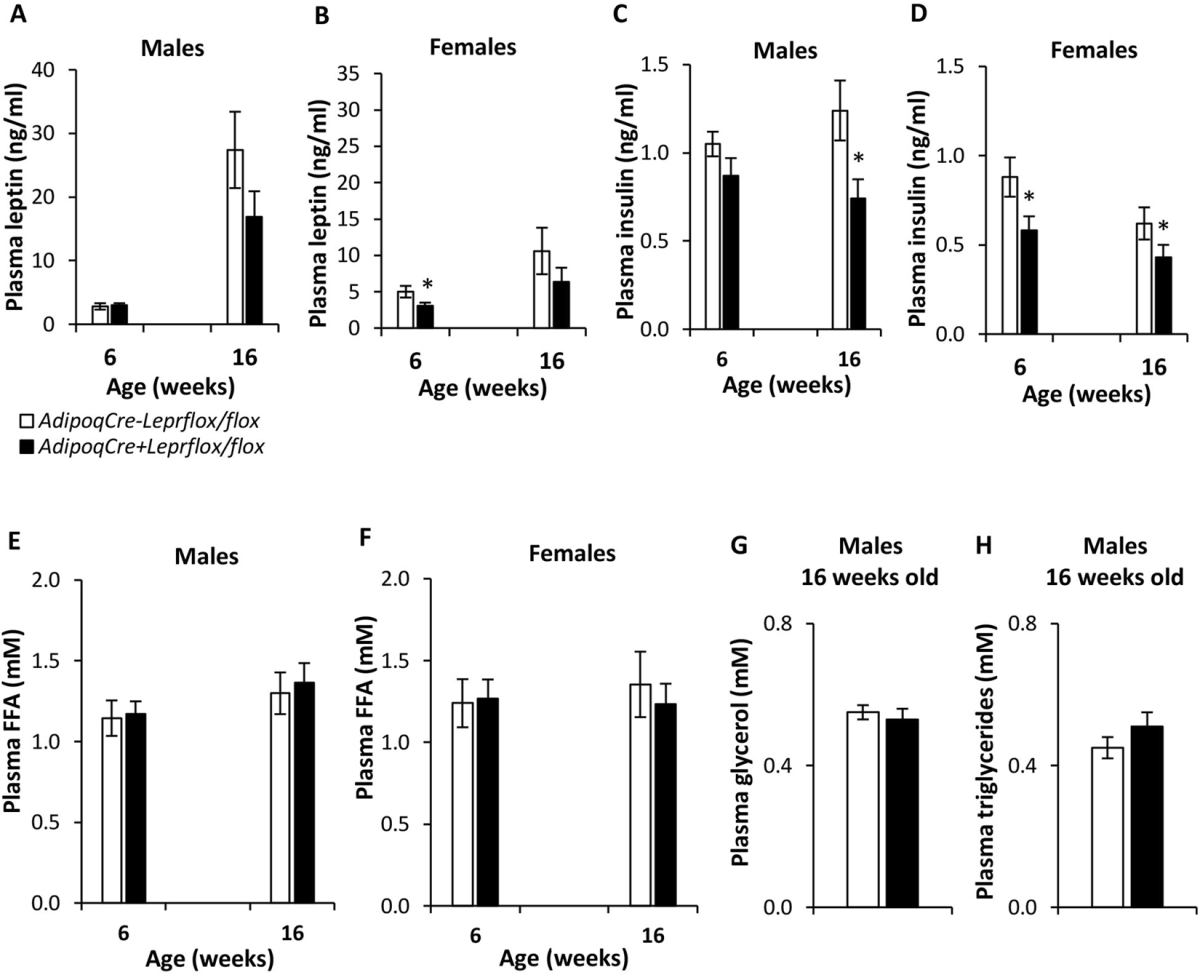
Plasma parameters at 6 and 16 weeks of age in male and female *AdipoqCre*^−^*Lepr*^*flox/flox*^ and *AdipoqCre*^+^*Lepr*^*flox/flox*^ mice. For males at 6 weeks of age, for *AdipoqCre*^−^*Lepr*^*flox/flox*^, n = 15 for leptin and n = 13 for insulin and for FFA; for *AdipoqCre*^+^*Lepr*^*flox/flox*^, n = 13 for leptin and for FFA and n = 11 for insulin. For females at 6 weeks of age, for *AdipoqCre*^−^*Lepr*^*flox/flox*^, n = 11 for leptin and for insulin, and n = 10 for FFA; for *AdipoqCre*^+^*Lepr*^*flox/flox*^, n = 13 for leptin, for insulin, and for FFA. For males at 16 weeks of age, for *AdipoqCre*^−^*Lepr*^*flox/flox*^, n = 15 for leptin, n = 14 for insulin and for FFA, and n = 12 for glycerol and for triglycerides; for *AdipoqCre*^+^*Lepr*^*flox/flox*^, n = 13 for leptin, n = 12 for insulin, n = 13 for FFA, and n = 11 for glycerol and for triglycerides. For females at 16 weeks of age, for *AdipoqCre*^−^*Lepr*^*flox/flox*^, n = 11 for leptin and n = 9 for insulin and for FFA; for *AdipoqCre*^+^*Lepr*^*flox/flox*^, n = 13 for leptin and n = 12 for insulin and for FFA. Plasma obtained from saphenous vein blood. For each sex, an unpaired t-test was performed for each parameter, except for male and female leptin and insulin at 16 weeks old as well as female FFA at 6 weeks old, where the Mann-Whitney *U* test was used. *p < 0.05 vs. *AdipoqCre*^−^*Lepr*^*flox/flox*^ of same sex, at each age.

Various plasma analytes of these mice were measured at 6 and 16 weeks of age (Fig. 3). At 6 weeks of age, plasma leptin levels did not significantly differ between male knockdown and control mice, but female knockdown mice had lower leptin concentrations than female controls (Fig. 3A,B). At 16 weeks of age, although plasma leptin concentrations did not differ between knockdown and control mice, the relationship between body weight and plasma leptin was weaker in knockdown mice than controls. Among males, Spearman correlation (ρ) was 0.854 (p < 0.05) for controls and 0.625 (p < 0.05) for knockdown mice. Among females, Spearman correlation was 0.827 (p < 0.05) for controls, whereas ρ was not significant in knockdown mice. At 6 weeks of age, plasma insulin levels did not significantly differ between genotypes in males, but female knockdown mice had lower insulin concentrations than controls. At 16 weeks of age, insulin was significantly lower in knockdown mice vs. control mice in males and females (Fig. 3C,D). Plasma FFAs in males and females, and plasma glycerol and TGs in males were not significantly different between genotypes (Fig. 3E–H).

**Figure 4:**
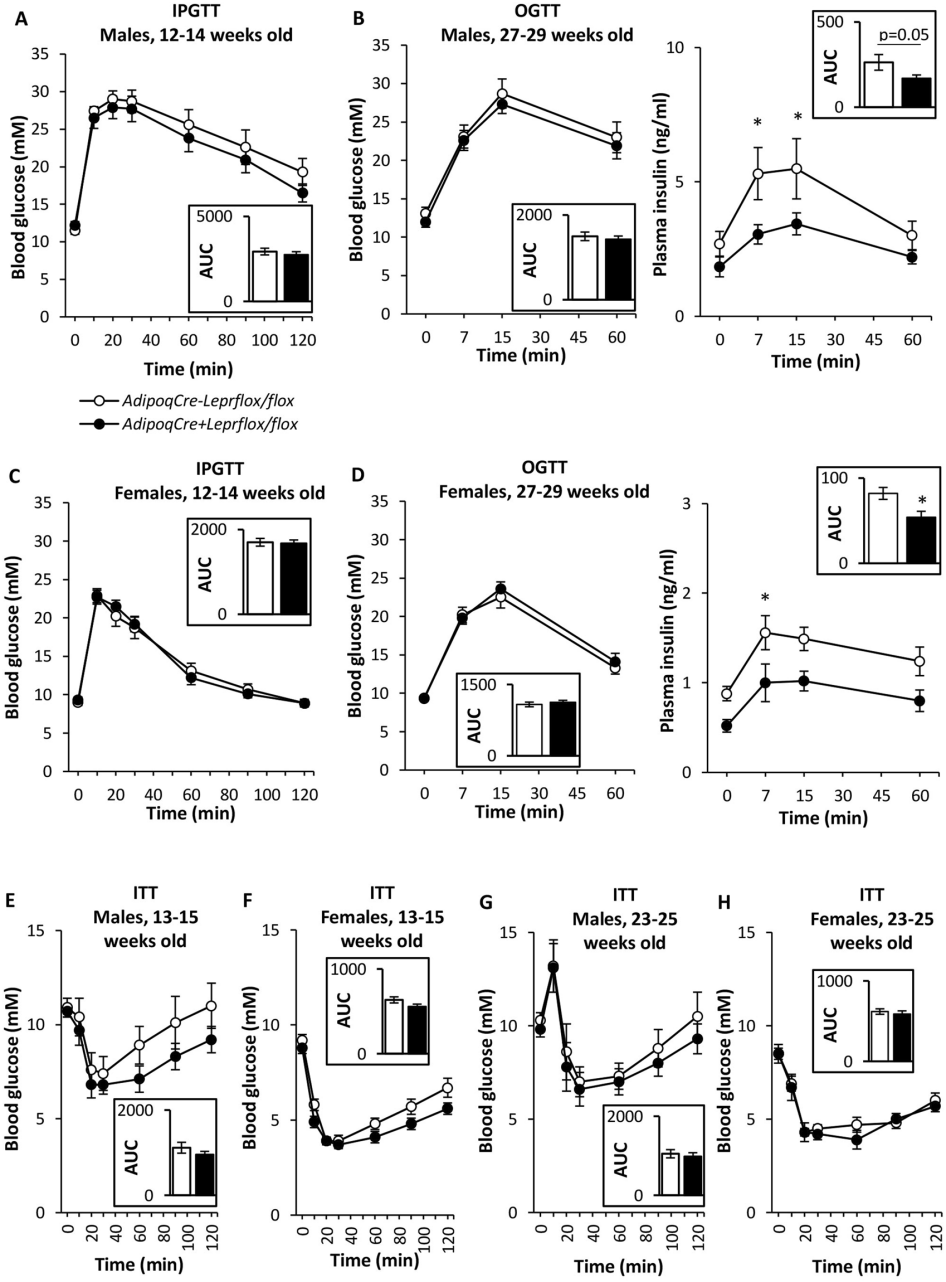
Metabolic tests in male and female *AdipoqCre*^−^*Lepr*^*flox/flox*^ and *AdipoqCre*^+^*Lepr*^*flox/flox*^. (**A**) IPGTT (1.5 g/kg) in males; n = 10 *AdipoqCre*^−^*Lepr*^*flox/flox*^ and n = 14 *AdipoqCre*^+^*Lepr*^*flox/flox*^. **(B)** Glucose (left) and insulin (right) during OGTT (1.5 g/kg) in males; n = 10 *AdipoqCre*^−^*Lepr*^*flox/flox*^ and n = 14 *AdipoqCre*^+^*Lepr*^*flox/flox*^. **(C)** IPGTT (1.5 g/kg) in females; n = 14 *AdipoqCre*^−^*Lepr*^*flox/flox*^ and n = 19 *AdipoqCre*^+^*Lepr*^*flox/flox*^. **(D)** Glucose (left) and insulin (right) during OGTT (1.5 g/kg) in females; n = 6 *AdipoqCre*^−^*Lepr*^*flox/flox*^ and n = 7 *AdipoqCre*^+^*Lepr*^*flox/flox*^. **(E)** ITT (0.65 U/kg) in males; n = 10 *AdipoqCre*^−^*Lepr*^*flox/flox*^ and n = 14 *AdipoqCre*^+^*Lepr*^*flox/flox*^. **(F)** ITT (0.65 U/kg) in females; n = 14 *AdipoqCre*^−^*Lepr*^*flox/flox*^ and n = 18 *AdipoqCre*^+^*Lepr*^*flox/flox*^. (**G**) ITT (0.75 U/kg) in males; n = 10 *AdipoqCre*^−^*Lepr*^*flox/flox*^ and n = 13 *AdipoqCre*^+^*Lepr*^*flox/flox*^. (**H**) ITT (0.75 U/kg) in females; n = 6 *AdipoqCre*^−^*Lepr*^*flox/flox*^ and n = 9 *AdipoqCre*^+^*Lepr*^*flox/flox*^. Repeated measures two-way ANOVA with Bonferroni *post-hoc* test was performed for parameters measured over time. An unpaired t-test was performed for each area under the curve (AUC) calculation, except for glucose concentration in (**D**), where Mann-Whitney *U* test was used. *p < 0.05 vs. *AdipoqCre*^−^*Lepr*^*flox/flox*^. In (**A**–**H**), there is a main effect of time (p < 0.05).

**Figure 5:**
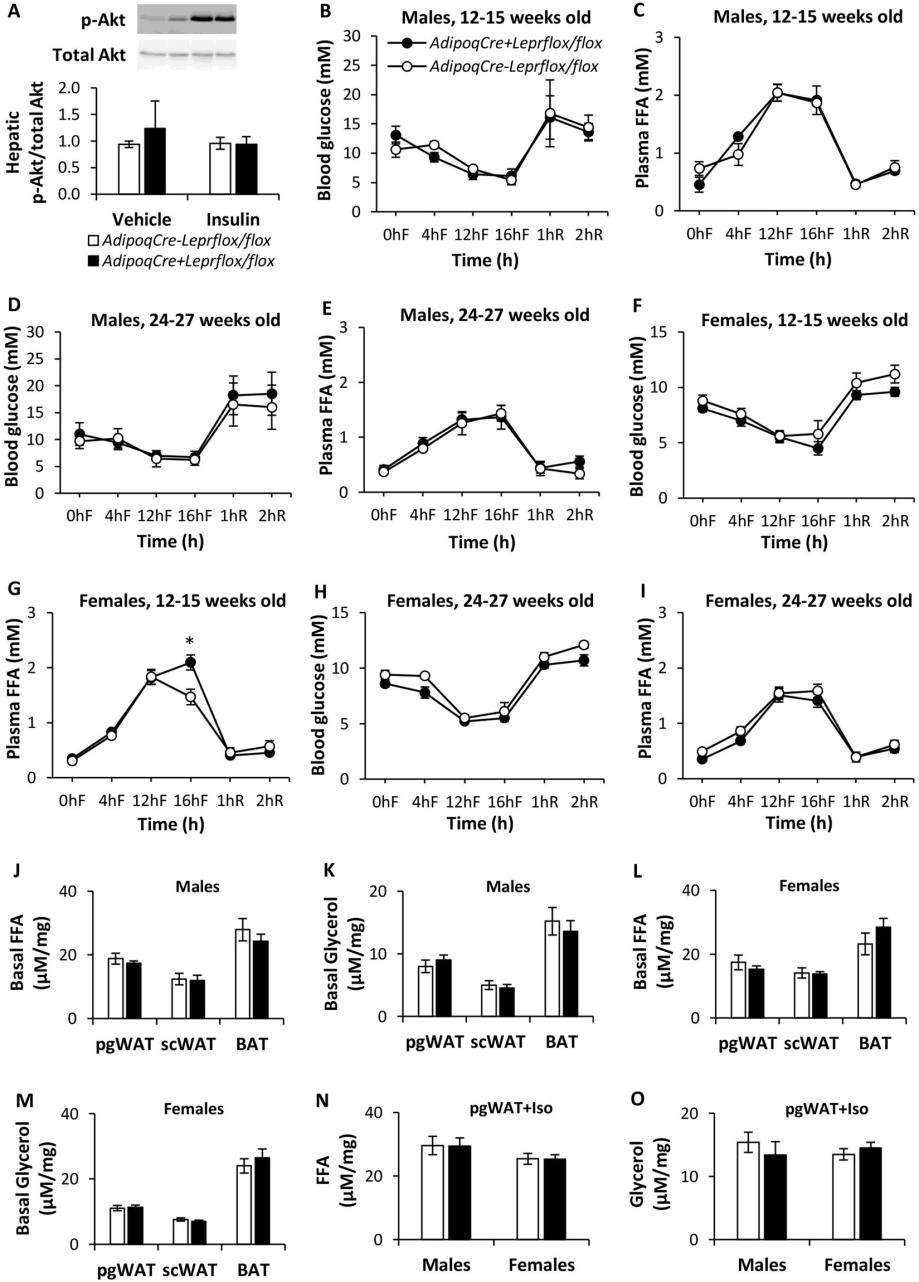
Effect of intraportal insulin or vehicle on the ratio of phospho-Akt (Ser473) to total Akt in the liver of female *AdipoqCre*^−^*Lepr*^*flox/flox*^ and *AdipoqCre*^+^*Lepr*^*flox/flox*^ mice (**A**). For vehicle injection, n = 3 per genotype, and for insulin injection, n = 4 per genotype. For each type of injection, quantification was normalized to bands of *AdipoqCre*^−^*Lepr*^*flox/flox*^ mice. Results for each protein are from 2 separate blots and Supplementary Fig. S4 contains the full-length blots. Representative blots for each protein are from the same samples in the first blot. Blood glucose and plasma FFA concentrations during fasting-refeeding experiments at different ages in male and female *AdipoqCre*^−^*Lepr*^*flox/flox*^ and *AdipoqCre*^+^*Lepr*^*flox/flox*^ mice (**B**–**I**). For younger males, n = 3 per genotype. For older males, n = 4 for *AdipoqCre*^−^*Lepr*^*flox/flox*^ and n = 5 for *AdipoqCre*^+^*Lepr*^*flox/flox*^. For younger females, n = 4 for *AdipoqCre*^−^*Lepr*^*flox/flox*^ and n = 6 for *AdipoqCre*^+^*Lepr*^*flox/flox*^. For older females, n = 6 for *AdipoqCre*^−^*Lepr*^*flox/flox*^ and n = 9 for *AdipoqCre*^+^*Lepr*^*flox/flox*^. FFA and glycerol concentrations in media collected in *ex vivo* lipolysis assay under basal (**J**–**M**) and isoprenaline-stimulated conditions (**N**,**O**). In (**J**,**K**), n = 6 for *AdipoqCre*^−^*Lepr*^*flox/flox*^ and n = 7 for *AdipoqCre*^+^*Lepr*^*flox/flox*^. In (**L**,**M**), n = 8 for *AdipoqCre*^−^*Lepr*^*flox/flox*^ and n = 9 for *AdipoqCre*^+^*Lepr*^*flox/flox*^. In (**N**,**O**), n = 6 per genotype for males and for females, n = 6 for *AdipoqCre*^−^*Lepr*^*flox/flox*^ and n = 8 for *AdipoqCre*^+^*Lepr*^*flox/flox*^. In (**A**), the Mann-Whitney *U* test was performed for vehicle injection and an unpaired t-test was performed insulin injection. In (**B**–**I**), repeated measures two-way ANOVA with Bonferroni *post-hoc* test was performed and there is a main effect of time (p < 0.05). In (**J**–**O**), an unpaired t-test was used to compare the two genotypes for each tissue and parameter. *p < 0.05 vs. *AdipoqCre*^−^*Lepr*^*flox/flox*^. F, fast; R, refed; Iso, isoprenaline; pgWAT, perigonadal white adipose tissue; scWAT, subcutaneous white adipose tissue.

Glucose tolerance assessed by IPGTT was similar between male knockdown mice and controls (Fig. 4A). IPGTTs provide a more direct assessment of the role of the pancreas in glucose metabolism, while OGTTs also include the incretin response^24^. At a later age, the blood glucose excursion during the OGTT was similar between male knockdown and control mice, but the knockdown mice had a lower insulin response (p < 0.05 at 7 and 15 min; Fig. 4B). Similar results were obtained in IPGTTs and OGTTs for females (Fig. 4C,D). Neither male nor female knockdown mice had statistically significant differences in insulin sensitivity, assessed by ITT, compared to their controls at different ages (Fig. 4E–H). Moreover, insulin was injected via the portal vein to assess phosphorylated Akt in the liver, a marker of hepatic insulin sensitivity. The ratio of insulin-stimulated phospho-Akt (Ser473) to total Akt in the liver was similar between Lepr knockdown and Flox control mice (Fig. 5A). The ratio was also similar between genotypes in vehicle-injected mice (Fig. 5A). During fasting, hepatic glycogen content was similar between Lepr knockdown and Flox control mice (2.29 ± 0.64 and 2.82 ± 0.41 mg per g liver, respectively; not statistically significant).

We performed fasting-refeeding experiments in both sexes at different ages (Fig. 5B–I) and found that blood glucose did not differ between knockdown and control mice, but younger female knockdown mice had higher plasma FFA concentrations after a prolonged fast (16 h) compared to controls (Fig. 5G). The rate of lipolysis, which is based on the amount of FFA and glycerol released *ex vivo* by different adipose tissue depots, was not altered in male and female knockdown mice compared to their controls (Fig. 5J–M). The response of pgWAT to isoprenaline, a β_3_-receptor adrenergic receptor agonist, was also comparable between knockdown mice and controls in both sexes (Fig. 5N,O). Leptin secretion from pgWAT and scWAT samples was similar between Lepr knockdown and Flox controls, among males and females (Supplementary Fig. S5).

To assess the contribution of adipose tissue leptin signalling to the glucose-lowering effect of exogenous leptin during insulin deficiency, male mice were rendered diabetic with STZ and subsequently treated with murine leptin delivered via mini-osmotic pumps for 8 days. While all mice had similar hyperglycemia following STZ injections, leptin therapy induced more rapid normalization in blood glucose levels in the knockdown mice (Supplementary Fig. S6). STZ + leptin groups had a progressive decrease in body weight vs. Sham controls (Supplementary Fig. S6). Mice became hypoleptinemic following STZ administration and leptin therapy increased plasma leptin levels to a greater extent in knockdown vs. Flox controls (p < 0.05, Supplementary Fig. S6). The elevated plasma leptin concentrations in the Lepr knockdown STZ + leptin group vs. Flox control STZ + leptin group were associated with increased concentrations of leptin receptor in plasma (p < 0.05, Supplementary Fig. S6). STZ-injected groups had similar insulin deficiency (Supplementary Fig. S6). To test if the duration of leptin therapy affected the difference in plasma leptin concentrations between genotypes among STZ-injected mice, we treated diabetic mice with a single injection of murine leptin and monitored their blood glucose and plasma leptin over 6 hours, a protocol similar to that used by Burnett *et al*. in non-diabetic mice^22^. The single leptin injection did not lower blood glucose differentially in diabetic Lepr knockdown vs. Flox control mice and plasma leptin levels were similar between genotypes at all timepoints (Supplementary Fig. S7). The half-life of leptin, with 30 min post-injection as the starting time for the calculation (time of highest average leptin concentration), was 53 ± 6 min for Lepr knockdown and 57 ± 12 min for Flox control mice, a difference that was not statistically significant.

### Mice with adipose tissue-specific *Lepr* reconstitution

**Figure 6:**
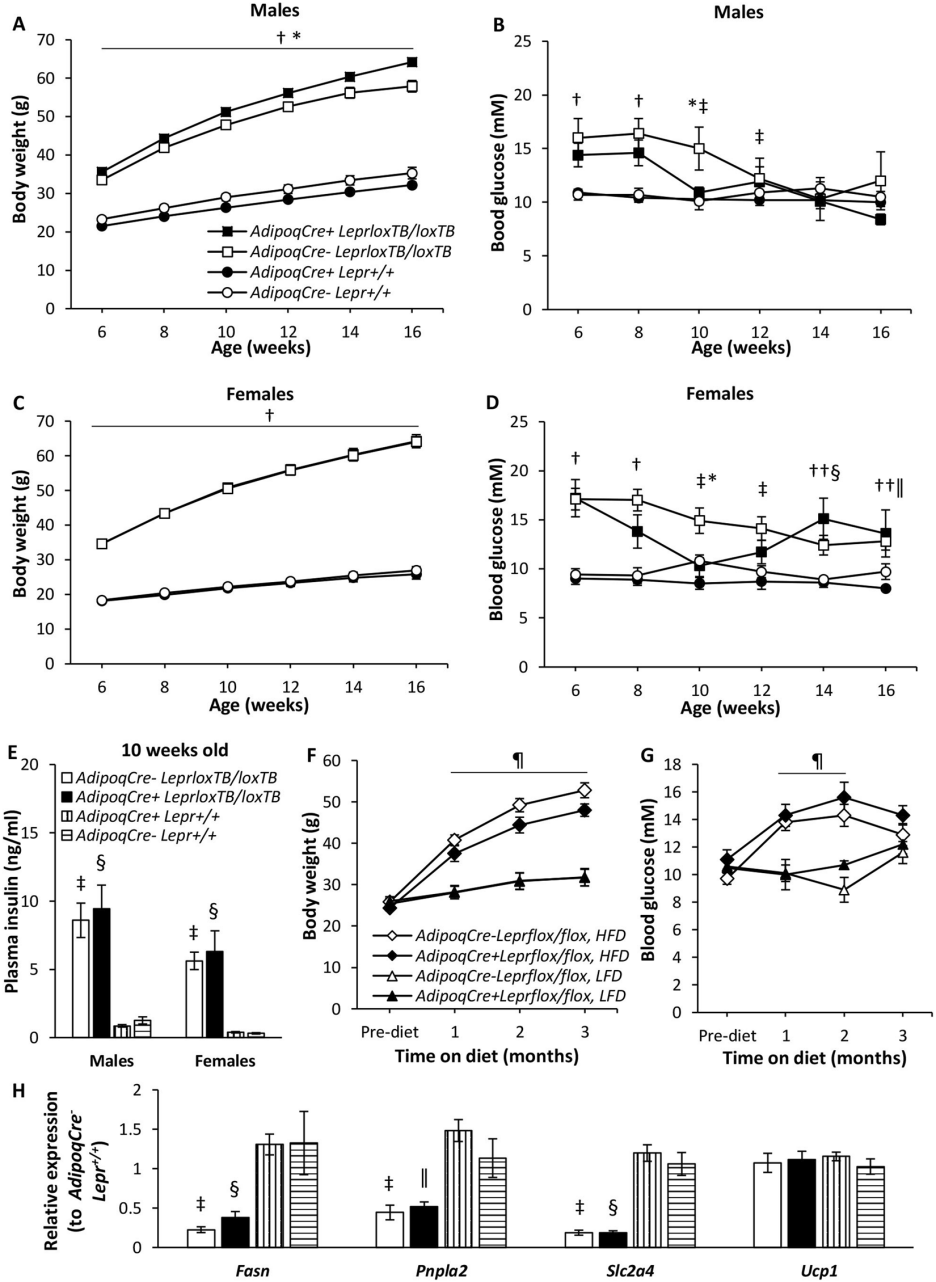
Body weight, blood glucose, and plasma insulin concentrations at 10 weeks old of male and female mice from the ATLeprEXP colony (**A**–**E**) and of male mice from the AdipoqKO colony on a high (HFD) or low fat diet (LFD) (**F**,**G**), obtained after a 4 h fast. In (**A**,**B**,**E**), n = 10 for *AdipoqCre*^+^*Lepr*^*loxTB/loxTB*^, except at 16 weeks of age, when n = 9; n = 11 for *AdipoqCre*^−^*Lepr*^*loxTB/loxTB*^, except at 16 weeks of age, when n = 9; n = 16 for *AdipoqCre*^+^*Lepr*^+/+^; n = 9 for *AdipoqCre*^−^*Lepr*^+/+^. In (**C**,**D**,**E**), n = 8 for *AdipoqCre*^+^*Lepr*^*loxTB/loxTB*^; n = 15 for *AdipoqCre*^−^*Lepr*^*loxTB/loxTB*^; n = 8 for *AdipoqCre*^+^*Lepr*^+/+^; n = 7 for *AdipoqCre*^−^*Lepr*^+/+^. In (**F**,**G)**, Pre-diet refers to the day before the start of diet; for *AdipoqCre*^−^*Lepr*^*flox/flox*^, n = 10 on HFD and n = 7 on LFD, and for *AdipoqCre*^+^*Lepr*^*flox/flox*^, n = 8 on HFD and n = 4 on LFD. In (**H**), RT-qPCR for *Fasn*, *Pnpla2*, and *Slc2a4* was done in perigonadal white adipose tissue and for *Ucp1* in brown adipose tissue; all samples were from male mice, 17–20 weeks old, from the ATLeprEXP colony. In (**H**), n = 8 for *AdipoqCre*^+^*Lepr*^*loxTB/loxTB*^, except for *Ucp1* where n = 9; n = 10 for *AdipoqCre*^−^*Lepr*^*flox/flox*^, except for *Ucp1* where n = 11; n = 14 for *AdipoqCre*^+^*Lepr*^+/+^, except for *Ucp1* where n = 15; and n = 7 for *AdipoqCre*^−^*Lepr*^+/+^, except for *Ucp1* where n = 8. For body weights of males and females from ATLeprEXP colony (**A**,**C**), only statistical significance of maximum body weight (asymptote) is indicated in the figure. In (**F**,**G**), repeated measures two-way ANOVA with Tukey *post-hoc* test was performed and there is a main effect of time (p < 0.05). In (**E**), for each sex, and in (**H**), for each parameter, one-way ANOVA with Tukey *post-hoc* was carried out. *p < 0.05, *AdipoqCre*^+^*Lepr*^*loxTB/loxTB*^ vs *AdipoqCre*^−^*Lepr*^*loxTB/loxTB*^; ^†^p < 0.05, *AdipoqCre*^+^*Lepr*^*loxTB/loxTB*^ and *AdipoqCre*^−^*Lepr*^*loxTB/loxTB*^ vs *AdipoqCre*^+^*Lepr*^+/+^ and *AdipoqCre*^−^*Lepr*^+/+^; ^‡^p < 0.05, *AdipoqCre*^−^*Lepr*^*loxTB/loxTB*^ vs *AdipoqCre*^+^*Lepr*^+/+^ and *AdipoqCre*^−^*Lepr*^+/+^; ^§^p < 0.05, *AdipoqCre*^+^*Lepr*^*loxTB/loxTB*^ vs *AdipoqCre*^+^*Lepr*^+/+^ and *AdipoqCre*^−^*Lepr*^+/+^; ^ǁ^p < 0.05, *AdipoqCre*^+^*Lepr*^*loxTB/loxTB*^ vs *AdipoqCre*^+^*Lepr*^+/+^. ^††^p < 0.05, *AdipoqCre*^−^*Lepr*^*loxTB/loxTB*^ vs. *AdipoqCre*^+^*Lepr*^+/+^. ^¶^p < 0.05, HFD groups vs. LFD groups.

As an alternative approach to understand the consequences of leptin signalling in adipose tissue, we investigated the effect of reconstituting Lepr expression specifically in adipose tissues of *Lepr*^*loxTB/loxTB*^ mice, in which global Lepr expression is inhibited due to a transcriptional block. These mice have a similar phenotype to *db/db* mice including obesity and hyperglycemia^35^. Male *Lepr*^*loxTB/loxTB*^ mice grow faster and reach a higher maximum body weight compared to controls (Fig. 6A and Supplementary Fig. S8), and interestingly, mice with Lepr expression selectively in adipose tissues (*AdipoqCre*^+^*Lepr*^*loxTB/loxTB*^) have a higher maximum body weight than mice lacking Lepr expression globally (*AdipoqCre*^−^*Lepr*^*loxTB/loxTB*^) (Fig. 6A). No differences in body composition, assessed by DEXA, were detected in male *AdipoqCre*^+^*Lepr*^*loxTB/loxTB*^ vs. *AdipoqCre*^−^*Lepr*^*loxTB/loxTB*^ mice, but as expected *Lepr*^*loxTB/loxTB*^ mice had higher body fat content compared to male controls (*AdipoqCre*^+^*Lepr*^+/+^ and *AdipoqCre*^−^*Lepr*^+/+^ mice) (Table 1). Although initially hyperglycemic, male *AdipoqCre*^+^*Lepr*^*loxTB/loxTB*^ mice had similar blood glucose levels to controls starting at 10 weeks of age, but this normalization was delayed in male *AdipoqCre*^−^*Lepr*^*loxTB/loxTB*^ mice (Fig. 6B). Although *Lepr*^*loxTB/loxTB*^ females were obese compared to controls, the maximum body weight of female *AdipoqCre*^+^*Lepr*^*loxTB/loxTB*^ mice did not differ from that of *AdipoqCre*^−^*Lepr*^*loxTB/loxTB*^ mice (Fig. 6C and Supplementary Fig. S8). Body composition was similar between female *AdipoqCre*^+^*Lepr*^*loxTB/loxTB*^ and *AdipoqCre*^−^*Lepr*^*loxTB/loxTB*^ mice (Table 1). At 6 weeks of age, *Lepr*^*loxTB/loxTB*^ females were hyperglycemic (Fig. 6D). *AdipoqCre*^+^*Lepr*^*loxTB/loxTB*^ female mice reached normal glucose levels by 10 weeks but both *AdipoqCre*^+^*Lepr*^*loxTB/loxTB*^ and *AdipoqCre*^−^*Lepr*^*loxTB/loxTB*^ female mice had slightly elevated blood glucose (p < 0.05 vs. controls) at 14 and 16 weeks of age. The improvement in glycemia among *AdipoqCre*^+^*Lepr*^*loxTB/loxTB*^ mice compared to *AdipoqCre*^−^*Lepr*^*loxTB/loxTB*^ mice at 10 weeks of age was not associated with statistically significant differences in plasma insulin concentrations between these two genotypes; they were both hyperinsulinemic (Fig. 6E). At an older age, mice with adipose tissue Lepr reconstitution had higher plasma insulin concentrations than mice with global inhibition of Lepr during the OGTT, despite having similar glucose concentrations throughout the test (Supplementary Fig. S9). These results are complementary to those obtained during the OGTT in the adipose tissue Lepr knockdown colony. Mice with adipose tissue Lepr reconstitution also had higher blood glucose at 120 min of the IPGTT compared to mice with global inhibition of Lepr and differences in the plasma insulin profile throughout IPGTT were not statistically significant (Supplementary Fig. S9).

**Table 1:** Body composition in mice from ATLeprEXP colony.

Sex	Genotype	Lean mass (g)	Fat mass (g)	Sum of lean and fat mass (g)	Percent fat
M	*AdipoqCre* ^+^ *Lepr* ^*loxTB/loxTB*^	25.9 ± 0.6^‖‡^	36.9 ± 0.8^†^	62.7 ± 1.0^†^	58.8 ± 0.7^†^
	*AdipoqCre* ^−^ *Lepr* ^*loxTB/loxTB*^	25.2 ± 0.5	33.7 ± 1.4^†^	58.8 ± 1.7^†^	57.0 ± 1.1^†^
	*AdipoqCre* ^+^ *Lepr* ^+/+^	23.2 ± 0.4	8.3 ± 0.7	31.5 ± 0.9	25.9 ± 1.4
	*AdipoqCre* ^−^ *Lepr* ^+/+^	24.1 ± 0.7	9.8 ± 1.2	33.8 ± 1.6	28.4 ± 2.5
F	*AdipoqCre* ^+^ *Lepr* ^*loxTB/loxTB*^	24.2 ± 1.4^†^	39.1 ± 1.3^†^	63.2 ± 2.1^†^	62.0 ± 1.4^†^
	*AdipoqCre* ^−^ *Lepr* ^*loxTB/loxTB*^	25.3 ± 0.5^†^	39.1 ± 0.7^†^	64.3 ± 0.9^†^	60.7 ± 0.5^†^
	*AdipoqCre* ^+^ *Lepr* ^+/+^	18.7 ± 0.6	6.7 ± 1.0	25.4 ± 1.5	25.5 ± 2.4
	*AdipoqCre* ^−^ *Lepr* ^+/+^	19.0 ± 0.7	7.6 ± 0.8	26.7 ± 1.4	28.1 ± 1.8

For males, n = 8 for *AdipoqCre*^−^*Lepr*^*loxTB/loxTB*^, n = 10 for *AdipoqCre*^+^*Lepr*^*loxTB/loxTB*^, n = 16 for *AdipoqCre*^+^*Lepr*^+/+^, n = 7 for *AdipoqCre*^−^*Lepr*^+/+^. For females, n = 15 for *AdipoqCre*^−^*Lepr*^*loxTB/loxTB*^, n = 8 for *AdipoqCre*^+^*Lepr*^*loxTB/loxTB*^, n = 8 for *AdipoqCre*^+^*Lepr*^+/+^, n = 7 for *AdipoqCre*^−^*Lepr*^+/+^. ^†^p < 0.05, *AdipoqCre*^+^*Lepr*^*loxTB/loxTB*^ and *AdipoqCre*^−^*Lepr*^*loxTB/loxTB*^ vs *AdipoqCre*^+^*Lepr*^+/+^ and *AdipoqCre*^−^*Lepr*^+/+^; ^‖^p < 0.05, *AdipoqCre*^+^*Lepr*^*loxTB/loxTB*^ vs *AdipoqCre*^+^*Lepr*^+/+^; ^‡^p < 0.05, *AdipoqCre*^−^*Lepr*^*loxTB/loxTB*^ vs *AdipoqCre*^+^*Lepr*^+/+^; ^§^p < 0.05, *AdipoqCre*^−^*Lepr*^*loxTB/loxTB*^ vs *AdipoqCre*^+^*Lepr*^+/+^ and *AdipoqCre*^−^*Lepr*^+/+^. For each parameter within each sex, one-way ANOVA followed by Tukey’s test was performed. Age = 17–19 weeks of age.

Lepr knockdown mice and Flox controls on HFD were similarly heavier relative to mice on LFD (Fig. 6F) and transient hyperglycemia was observed in both genotypes (Fig. 6G). Interestingly, the magnitude of reduced weight gain in male mice with disruption of *Leprb* in adipose tissue was similar to that of the increased weight gain in male mice with reconstitution of *Leprb* in adipose tissue.

Expression of enzymes involved in lipid synthesis (*Fasn*) and lipolysis (*Pnpla2*), and of the glucose transporter GLUT4 (*Slc2a4*) was also assessed in pgWAT of male mice from the ATLeprEXP colony (Fig. 6G). Their expression was decreased in *Lepr*^*loxTB/loxTB*^ mice compared to controls (p < 0.05), but statistically significant differences were not detected between *AdipoqCre*^+^*Lepr*^*loxTB/loxTB*^ and *AdipoqCre*^−^*Lepr*^*loxTB/loxTB*^ mice. The expression of *Ucp1* in BAT of males was similar among the 4 genotypes (Fig. 6H).

**Figure 7:**
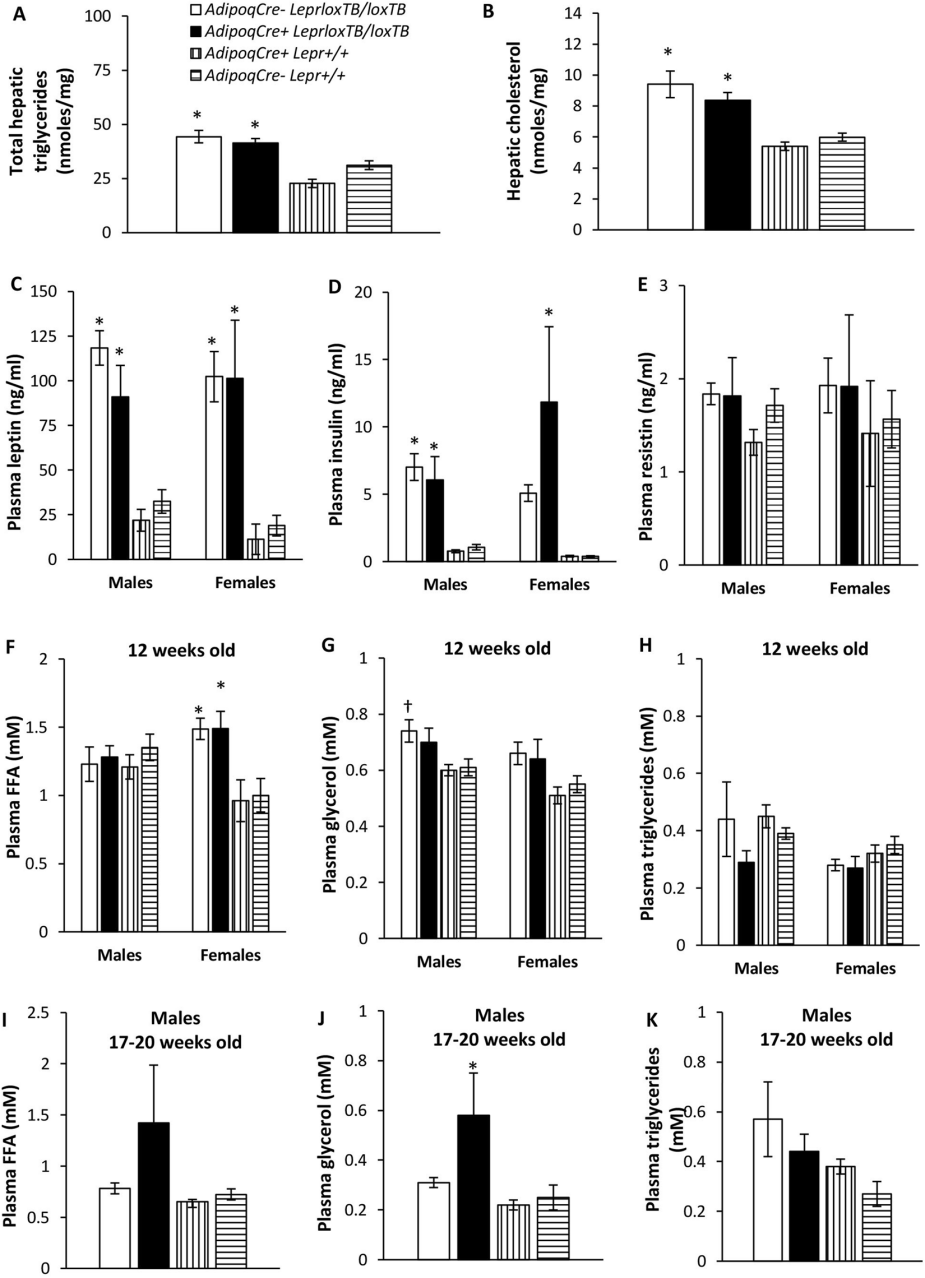
Hepatic (**A**,**B**) and plasma (**C**–**K**) parameters of mice from the ATLeprEXP colony. Liver samples used for the analysis in (**A**,**B**) were obtained from male mice at the time of euthanasia (17–20 weeks old). In (**A**,**B**), n = 10 for *AdipoqCre*^−^*Lepr*^*loxTB/TB*^, n = 9 for *AdipoqCre*^+^*Lepr*^*loxTB/TB*^, n = 16 for *AdipoqCre*^+^*Lepr*^+/+^, and n = 8 for *AdipoqCre*^−^*Lepr*^+/+^. Plasma obtained from cardiac blood collected at the time of euthanasia (17–20 weeks old) in (**C**–**E**) and (**I**–**K**), and from saphenous vein blood at 12 weeks old (**F**–**H**). For males in (**C**–**E**) and (**I**–**K**), n = 9 for *AdipoqCre*^−^*Lepr*^*loxTB/TB*^, n = 8 for *AdipoqCre*^+^*Lepr*^*loxTB/TB*^, n = 14 for *AdipoqCre*^+^*Lepr*^+/+^, n = 7 for *AdipoqCre*^−^*Lepr*^+/+^, except in (**I**–**K**), where n = 10 for *AdipoqCre*^−^*Lepr*^*loxTB/loxTB*^. For females in (**C**–**E**), n = 11 for *AdipoqCre*^−^*Lepr*^*loxTB/loxTB*^, n = 4 for *AdipoqCre*^+^*Lepr*^*loxTB/loxTB*^, n = 4 for *AdipoqCre*^+^*Lepr*^+/+^, and n = 5 for *AdipoqCre*^−^*Lepr*^+/+^. In (**F**–**H**), for males, n = 11 for *AdipoqCre*^−^*Lepr*^*loxTB/TB*^, n = 10 for *AdipoqCre*^+^*Lepr*^*loxTB/TB*^, n = 16 for *AdipoqCre*^+^*Lepr*^+/+^, n = 9 for *AdipoqCre*^−^*Lepr*^+/+^; for females, n = 15 for *AdipoqCre*^−^*Lepr*^*loxTB/TB*^, n = 8 for *AdipoqCre*^+^*Lepr*^*loxTB/TB*^, n = 8 for *AdipoqCre*^+^*Lepr*^+/+^, n = 7 for *AdipoqCre*^−^*Lepr*^+/+^. For each sex, one-way ANOVA followed by Tukey’s test was performed at each timepoint. *p < 0.05 vs. *AdipoqCre*^+^*Lepr*^+/+^ and *AdipoqCre*^−^*Lepr*^+/+^. ^†^p < 0.05 vs. *AdipoqCre*^+^*Lepr*^+/+^.

Levels of total triglycerides and cholesterol in the liver were higher in male *Lepr*^*loxTB/loxTB*^ mice compared to controls (p < 0.05), but were similar between *AdipoqCre*^+^*Lepr*^*loxTB/loxTB*^ and *AdipoqCre*^−^*Lepr*^*loxTB/loxTB*^ mice (Fig. 7A,B). *Lepr*^*loxTB/loxTB*^ male and female mice were hyperleptinemic (p < 0.05 vs. controls; Fig. 7C) and hyperinsulinemic (for males, p < 0.05 vs. controls; for females, p < 0.05 for *AdipoqCre*^+^*Lepr*^*loxTB/loxTB*^ vs. controls) (Fig. 7D). Plasma resistin was similar between groups (Fig. 7E). At 12 weeks of age, plasma FFA concentrations were not significantly different among males, but female *Lepr*^*loxTB/loxTB*^ mice had higher FFA concentrations compared to controls (p < 0.05; Fig. 7F). Plasma glycerol concentrations were higher in male *AdipoqCre*^−^*Lepr*^*loxTB/loxTB*^ mice compared to *AdipoqCre*^+^*Lepr*^+/+^ mice (p < 0.05), but no differences were found among female mice (Fig. 7G). Plasma triglycerides were similar among the 4 genotypes, regardless of sex (Fig. 7H). At a later age, in males, plasma FFAs and triglycerides were similar among the 4 genotypes, but plasma glycerol levels were 87% higher in male *AdipoqCre*^+^*Lepr*^*loxTB/loxTB*^ vs. *AdipoqCre*^−^*Lepr*^*loxTB/loxTB*^ mice (p < 0.05 for *AdipoqCre*^+^*Lepr*^*loxTB/loxTB*^ vs. controls; Fig. 7I–K).

## Discussion

We found that Cre-mediated excision of Lepr in adipose tissues modestly decreased body weight and diminished the insulin response during an oral glucose challenge in male and female mice. In the setting of insulin-deficiency, male mice with Lepr knockdown also had an accelerated glucose-lowering response to prolonged leptin administration compared to controls that was associated with increased concentrations of plasma leptin and leptin receptor. The half-life of leptin, calculated after a single injection of leptin, was similar between diabetic Lepr knockdown and Flox control mice. In complementary studies, reconstitution of Lepr selectively in adipose tissues of *Lepr*^*loxTB/loxTB*^ mice, which are obese and hyperglycemic, resulted in lower concentrations of blood glucose in both male and female mice at younger ages, and a greater maximum body weight was observed in male mice. Mice with reconstitution of Lepr in adipose tissues were also characterized by elevated plasma insulin concentrations during the OGTT and increased blood glucose concentrations during the IPGTT compared to mice with global inhibition of Lepr expression.

Cre-mediated recombination of *Lepr* was similar between males and females and in the Lepr knockdown and reconstitution studies. We observed relatively modest recombination using *AdipoqCre* mice, unlike most other reports. Cre efficiency can vary depending upon the floxed line^16^, but our results were comparable between *Lepr*^*flox/flox*^ and *Lepr*^*loxTB/loxTB*^ lines and the *mTmG* reporter line. Cre is expected to be expressed only in mature adipocytes^36^ and adipose tissue also contains immature adipocytes so perhaps the ratio of these cells, and ultimately the activity of the adiponectin promoter, is different in our lines compared to those that others have used.

Adiponectin and Lepr are both expressed in intestinal epithelium^30–33^. Tavernier *et al*.^31^ generated intestinal epithelial cell-specific Leprb knockout mice and achieved robust recombination in intestinal mucosa. A key finding in the paper was that following 12 weeks on high fat diet, intestinal epithelial cell-specific Leprb knockout mice were ~3 g lighter compared to Flox controls as a result of impaired nutrient absorption. Although after 12 weeks on high fat diet our mice with adipose tissue Lepr knockdown were ~5 g lighter vs Flox controls, we did not find evidence of Lepr recombination in intestinal mucosa. Therefore, we do not believe that Lepr recombination in the intestines is a major cause of the phenotype we observed. However, plasma leptin was lower in female adipose tissue Lepr knockdown mice at 6 weeks old, and at 16 weeks old there was a trend, albeit not statistically significant, for plasma leptin to be lower in male and female adipose tissue Lepr knockdown mice compared to controls. Therefore, it is possible that small changes in circulating leptin in mice with adipose tissue Lepr knockdown impaired absorption of nutrients, thereby reducing weight gain.

The results we obtained by knockdown of Lepr in adipose tissues contrast with results using antisense RNA to downregulate expression of leptin receptors in WAT^13^. In the study by Huan *et al*.^13^ male and female mice were characterized by obesity, glucose intolerance, and insulin resistance, although obesity and glucose intolerance appeared at a later age in females. We suggest four possible explanations for the different findings. First, we targeted Leprb while Huan *et al*.^13^ targeted all isoforms of Lepr, although downregulation was only reported for the short isoforms. The short isoforms predominate in WAT, but Leprb is considered to be responsible for the majority of the effects of leptin^13^. Second, we targeted BAT and WAT, while Huan *et al*. only targeted WAT. Ultimately, the phenotype of our mice is the net effect of alterations in Lepr signalling in BAT and WAT. However, we did not detect differences in the histology of BAT or in the *ex vivo* release of FFA and glycerol from BAT in Lepr knockdown mice vs. controls. Moreover, *Ucp1* expression was not altered in BAT of males with Lepr reconstitution. Third, while we used Cre-lox methodology, Huan *et al*. downregulated the expression of Lepr isoforms using antisense RNA expressed postnatally under the control of the PEPCK promoter. This knockdown approach has been suggested to induce obesity, regardless of the target RNA^14^. Finally, we can not discount the possibility that developmental effects in either model contribute to the observed differences.

Circulating hyperleptinemia was associated with the obese phenotype obtained by downregulation of leptin receptors in WAT using antisense RNA^13^. Hyperleptinemia was also observed upon reduction of Leprb in multiple peripheral tissues using a tamoxifen-inducible model, despite the mice being of normal body weight^11^. The differences in circulating levels of leptin in our mice with adipose tissue-specific knockdown of Lepr compared to control mice were not statistically significant, except in females at 6 weeks of age, when it was lower in Lepr knockdown mice. Moreover, using an *ex vivo* assay, leptin secretion by WAT samples, which was in the range of what has been reported^11,37^, was similar between Lepr knockdown and Flox control mice, in males and females. These findings do not support a negative feedback loop of leptin secretion via Leprb at the level of adipose tissues. However, we achieved modest recombination in adipose tissues and the short isoforms of Lepr may modulate leptin secretion and/or clearance, since Lepra knockout mice are characterized by higher levels of plasma leptin at 14 weeks old, but not at a later age^38^.

During the OGTT, mice with adipose tissue Lepr knockdown had lower plasma insulin concentrations than controls. Complementary results were obtained in mice with adipose tissue Lepr reconstitution, which had higher plasma insulin concentrations during the OGTT. The cause of the altered plasma insulin responses during the OGTT is unclear, but one possibility is that Lepr knockdown or reconstitution in adipose tissues alters the expression of adipokines that modulate glucose-stimulated insulin secretion. For instance, leptin can increase the secretion of interleukin-6 (IL-6)^39^, which in turn stimulates insulin secretion^40^. Crosstalk between adipose tissue and the pancreas may also explain the decrease in plasma insulin concentrations in male and female Lepr knockdown mice at 16 weeks old. Despite the reduced or augmented insulin response during the OGTT for Lepr knockdown or Lepr reconstitution mice, respectively, blood glucose concentrations were similar to those of controls throughout the OGTT and the underlying mechanism is unclear. Glucose tolerance is determined by insulin sensitivity and concentrations as well as insulin-independent stimulation of glucose disposal and inhibition of hepatic glucose production^41,42^. We did not detect significant differences in insulin sensitivity between Lepr knockdown and Flox controls by ITTs at different ages and insulin-stimulated hepatic Akt activation. Moreover, we found similar hepatic glycogen content in Lepr knockdown and Flox control mice during fasting, which suggests that the ability of glucose to promote its own disposal and storage is not improved in the liver of mice with Lepr knockdown. Lastly, during an IPGTT, while Lepr knockdown mice had similar blood glucose concentrations to controls, mice with Lepr reconstitution were mildly glucose intolerant. Our results indicate that adipose tissue leptin signalling does not greatly affect the blood glucose excursion following a glucose challenge.

Our results suggest that knockdown or reconstitution of Lepr in adipose tissues does not greatly affect lipid turnover. We did not find differences in adipose tissue lipolysis in Lepr knockdown mice vs. controls and the response of pgWAT to isoprenaline was also similar, suggesting that β_3_-adrenergic receptor signalling is intact in adipose tissues of Lepr knockdown mice. The concentration of lipids in plasma was also comparable between Lepr knockdown and Flox control mice. Male mice with adipose tissue-specific reconstitution of Lepr had plasma levels of FFAs and glycerol at 17–20 weeks of age that were approximately twice as high as the values in *AdipoqCre*^−^*Lepr*^*loxTB/loxTB*^ mice, consistent with leptin having a direct lipolytic effect on adipocytes^43^. However, lipid content in the liver, an organ that often accumulates lipids when adipose tissue lipolysis is elevated, was not higher in mice with Lepr reconstitution at the same age. Lepr reconstitution in males was without effect on the expression of mediators of lipid and glucose metabolism in pgWAT or on *Ucp1* expression in BAT.

Although most studies indicate that the CNS is the key tissue upon which peripherally administered leptin initially acts to trigger its glucose-lowering effects in insulin-deficient diabetes, it remains unclear if the CNS pathways involved are the same as for centrally administered leptin^44–48^. Perry *et al*.^49^ demonstrated that peripheral leptin diminishes hypothalamic-pituitary-adrenal (HPA) axis activity to reduce gluconeogenesis and glycemia, but this mechanism is specific to conditions of severe insulin deficiency because an increase in the magnitude of residual levels in insulin-deficient diabetes diminishes the ability of leptin to lower circulating glucose^50,51^. Herein, prolonged (8 days) leptin therapy resulted in a faster decrease in blood glucose in diabetic mice with adipose tissue-specific Lepr knockdown vs. controls and this was associated with significantly higher leptin levels following leptin therapy. Since endogenous plasma leptin levels were similar between genotypes in mice with normal levels of insulin (ie. not injected with STZ), leptin clearance may be impaired in insulin-deficient Lepr knockdown mice upon administration of prolonged exogenous leptin. The binding of circulating leptin to its leptin receptor diminishes the clearance of leptin^52^ and hence, the elevated concentration of plasma leptin receptor we observed in diabetic Lepr knockdown mice may contribute to the increased circulating leptin concentration in Lepr knockdown mice following 8 days of leptin therapy. However, the differential effect of exogenous leptin on plasma leptin concentrations in diabetic Lepr knockdown and Flox controls depends on the duration of leptin therapy. Following an acute injection of leptin, STZ-injected mice of both genotypes had similar plasma leptin profiles and leptin half-lives. After the single leptin injection, blood glucose profiles were similar between the two groups and, as expected based on our previous studies, blood glucose was not normalized^21^.

POMC neuron-specific expression of Lepr reduces obesity in *Lepr*^*loxTB/loxTB*^ males starting at 13 weeks of age, plateauing at a ~10 g differential at 15–20 weeks of age^8^. In our study, the magnitude of the difference in body weight between mice with adipose tissue-specific Lepr reconstitution (*AdipoqCre*^+^*Lepr*^*loxTB/loxTB*^) and loxTB control mice (*AdipoqCre*^−^*Lepr*^*loxTB/loxTB*^) was similar, ~6 g at 16 weeks of age, but Lepr reconstitution did not reduce obesity in our study. Our results indicate that obesity can be modestly prevented or augmented if Lepr expression is reduced or reconstituted, respectively, in adipose tissues of males. There were no differences in body weight between female mice with reconstitution of Lepr specifically in POMC neurons (*POMC-Cre*^+^*Lepr*^*loxTB/loxTB*^) and loxTB control mice (*POMC-Cre*^−^*Lepr*^*loxTB/loxTB*^)^8^. In our study, we also did not find an effect on body weight in females since the maximal body weight of female mice with adipose tissue-specific reconstitution of Lepr did not differ from that of female loxTB control mice.

Mice with Lepr reconstitution had lower blood glucose concentrations at certain ages, compared to *AdipoqCre*^−^*Lepr* ^*loxTB/loxTB*^ mice. The transient alterations in blood glucose concentration of *Lepr*^*loxTB/loxTB*^ male and female mice observed in our study are consistent with what has been reported in *db/db* mice, where blood glucose concentrations are inversely associated with plasma insulin concentrations^35^. The improved blood glucose concentrations in mice with adipose tissue Lepr reconstitution at younger ages was not due to elevations in plasma insulin and at older ages, mice with Lepr reconstitution were mildly glucose intolerant.

High fat diet diminishes Leprb expression in WAT and overexpression of Leprb in WAT and BAT, under the control of an aP2 promoter, inhibits the body weight gain in high fat diet-fed mice^15^. In contrast, we found that disruption of Lepr signalling in fat reduced body weight gain, and reconstitution of leptin signalling in fat in male mice lacking leptin signaling elsewhere increased weight gain. Although it is difficult to reconcile these results, the *aP2-Cre* model can promote recombination in both adipose and non-adipose tissues^16^.

In conclusion, our results indicate that peripheral leptin signalling in adipose tissues affects body weight regulation and glucose metabolism. Mice with adipose tissue-specific Lepr knockdown were modestly lighter, while reconstitution of Lepr in adipose tissues made male *Lepr*^*loxTB/loxTB*^ mice, which were obese, attain an even greater maximal body weight. In response to an oral glucose challenge, while mice with adipose tissue Lepr knockdown had a blunted plasma insulin profile, mice with adipose tissue Lepr reconstitution had higher plasma insulin concentrations compared to controls. Although mice with Lepr reconstitution, which were hyperleptinemic and hyperinsulinemic, had lower blood glucose compared to *AdipoqCre*^−^*Lepr*^*loxTB/loxTB*^ mice at certain younger ages, they were mildly glucose intolerant at older ages. In insulin-deficient diabetes, prolonged exogenous leptin therapy induced a faster reduction in blood glucose, possibly as a result of diminished leptin clearance. Additional research is warranted to further investigate the underlying mechanisms by which adipose tissue leptin signalling affects body weight and glucose homeostasis.

## Supplementary information

Supplementary Information

## Data Availability

The datasets generated during and/or analysed during the current study are available from the corresponding author on reasonable request.

## References

[1] Scott MM, Williams KW, Rossi J, Lee CE, Elmquist JK (2011). Leptin receptor expression in hindbrain Glp-1 neurons regulates food intake and energy balance in mice. Journal of Clinical Investigation.

[2] Dodd GT (2014). The thermogenic effect of leptin is dependent on a distinct population of prolactin-releasing peptide neurons in the dorsomedial hypothalamus. Cell Metab.

[3] Minokoshi Y, Haque MS, Shimazu T (1999). Microinjection of leptin into the ventromedial hypothalamus increases glucose uptake in peripheral tissues in rats. Diabetes.

[4] Bonzon-Kulichenko E (2009). Central leptin regulates total ceramide content and sterol regulatory element binding protein-1C proteolytic maturation in rat white adipose tissue. Endocrinology.

[5] Balthasar N (2004). Leptin receptor signaling in POMC neurons is required for normal body weight homeostasis. Neuron.

[6] van de Wall E (2008). Collective and individual functions of leptin receptor modulated neurons controlling metabolism and ingestion. Endocrinology.

[7] Shi H (2008). Sexually different actions of leptin in proopiomelanocortin neurons to regulate glucose homeostasis. Am J Physiol Endocrinol Metab.

[8] Berglund ED (2012). Direct leptin action on POMC neurons regulates glucose homeostasis and hepatic insulin sensitivity in mice. Journal of Clinical Investigation.

[9] Bates SH, Myers MG (2003). The role of leptin receptor signaling in feeding and neuroendocrine function. Trends Endocrinol Metab.

[10] Moon HS (2013). Leptin’s role in lipodystrophic and nonlipodystrophic insulin-resistant and diabetic individuals. Endocr Rev.

[11] Guo K (2007). Disruption of peripheral leptin signaling in mice results in hyperleptinemia without associated metabolic abnormalities. Endocrinology.

[12] Huynh FK (2010). Disruption of hepatic leptin signaling protects mice from age- and diet-related glucose intolerance. Diabetes.

[13] Huan JN (2003). Adipocyte-selective reduction of the leptin receptors induced by antisense RNA leads to increased adiposity, dyslipidemia, and insulin resistance. J Biol Chem.

[14] de Luca C (2005). Complete rescue of obesity, diabetes, and infertility in db/db mice by neuron-specific LEPR-B transgenes. Journal of Clinical Investigation.

[15] Wang MY, Orci L, Ravazzola M, Unger RH (2005). Fat storage in adipocytes requires inactivation of leptin’s paracrine activity: implications for treatment of human obesity. Proc Natl Acad Sci USA.

[16] Lee KY (2013). Lessons on conditional gene targeting in mouse adipose tissue. Diabetes.

[17] Eguchi J (2011). Transcriptional control of adipose lipid handling by IRF4. Cell Metab.

[18] McMinn JE (2004). An allelic series for the leptin receptor gene generated by CRE and FLP recombinase. Mamm Genome.

[19] Tuduri E (2014). Partial ablation of leptin signaling in mouse pancreatic alpha-cells does not alter either glucose or lipid homeostasis. Am J Physiol Endocrinol Metab.

[20] Muzumdar MD, Tasic B, Miyamichi K, Li L, Luo L (2007). A global double-fluorescent Cre reporter mouse. Genesis.

[21] Denroche HC (2015). Leptin induces fasting hypoglycaemia in a mouse model of diabetes through the depletion of glycerol. Diabetologia.

[22] Burnett LC, Skowronski AA, Rausch R, LeDuc CA, Leibel RL (2017). Determination of the half-life of circulating leptin in the mouse. Int J Obes (Lond).

[23] Denroche HC (2011). Leptin therapy reverses hyperglycemia in mice with streptozotocin-induced diabetes, independent of hepatic leptin signaling. Diabetes.

[24] Ayala JE (2010). Standard operating procedures for describing and performing metabolic tests of glucose homeostasis in mice. Dis.

[25] Lee KT, Karunakaran S, Ho MM, Clee SM (2011). PWD/PhJ and WSB/EiJ mice are resistant to diet-induced obesity but have normal insulin secretion. Endorinology.

[26] Ramey G, Faye A, Durel B, Viollet B, Vaulont S (2007). Iron overload in Hepc1(−/−) mice is not impairing glucose homeostasis. FEBS Lett.

[27] Sebastian D (2012). Mitofusin 2 (Mfn2) links mitochondrial and endoplasmic reticulum function with insulin signaling and is essential for normal glucose homeostasis. Proc Natl Acad Sci USA.

[28] Aljanabi SM, Martinez I (1997). Universal and rapid salt-extraction of high quality genomic DNA for PCR-based techniques. Nucleic Acids Research.

[29] Ruan H, Zarnowski MJ, Cushman SW, Lodish HF (2003). Standard isolation of primary adipose cells from mouse epididymal fat pads induces inflammatory mediators and down-regulates adipocyte genes. J Biol Chem.

[30] Iqbal J (2010). An intrinsic gut leptin-melanocortin pathway modulates intestinal microsomal triglyceride transfer protein and lipid absorption. J Lipid Res.

[31] Tavernier A (2014). Intestinal deletion of leptin signaling alters activity of nutrient transporters and delayed the onset of obesity in mice. Faseb J.

[32] Higurashi T (2014). Conditional knockout of the leptin receptor in the colonic epithelium revealed the local effects of leptin receptor signaling in the progression of colonic tumors in mice. Carcinogenesis.

[33] Su X (2015). Expression of FABP4, adipsin and adiponectin in Paneth cells is modulated by gut Lactobacillus. Sci.

[34] Sakaguchi M (2017). Adipocyte dynamics and reversible metabolic syndrome in mice with an inducible adipocyte-specific deletion of the insulin receptor. Cell Metabolism.

[35] Davis RC (2010). Early hepatic insulin resistance precedes the onset of diabetes in obese C57BLKS-db/db mice. Diabetes.

[36] Berry R, Rodeheffer MS (2013). Characterization of the adipocyte cellular lineage *in vivo*. Nature Cell Biology.

[37] Lee MJ, Fried SK (2006). Multilevel regulation of leptin storage, turnover, and secretion by feeding and insulin in rat adipose tissue. J Lipid Res.

[38] Li Z, Ceccarini G, Eisenstein M, Tan K, Friedman JM (2013). Phenotypic effects of an induced mutation of the ObRa isoform of the leptin receptor. Mol Metab.

[39] Yang WH (2013). Leptin induces IL-6 expression through OBRl receptor signaling pathway in human synovial fibroblasts. PLoS ONE.

[40] Dunmore SJ, Brown JE (2013). The role of adipokines in beta-cell failure of type 2 diabetes. J Endocrinol.

[41] Best JD (1996). Role of glucose effectiveness in the determination of glucose tolerance. Diabetes Care.

[42] Pacini G, Thomaseth K, Ahren B (2001). Contribution to glucose tolerance of insulin-independent vs. insulin-dependent mechanisms in mice. Am J Physiol Endocrinol Metab.

[43] Harris RB (2014). Direct and indirect effects of leptin on adipocyte metabolism. Biochim Biophys Acta.

[44] Perry RJ, Petersen KF, Shulman GI (2016). Pleotropic effects of leptin to reverse insulin resistance and diabetic ketoacidosis. Diabetologia.

[45] Meek TH, Morton GJ (2016). The role of leptin in diabetes: metabolic effects. Diabetologia.

[46] Fujikawa T (2013). Leptin engages a hypothalamic neurocircuitry to permit survival in the absence of insulin. Cell Metab.

[47] Xu Y, Chang JT, Myers MG, Xu Y, Tong Q (2016). Euglycemia Restoration by Central Leptin in Type 1 Diabetes Requires STAT3 Signaling but Not Fast-Acting Neurotransmitter Release. Diabetes.

[48] Xu J (2018). Genetic identification of leptin neural circuits in energy and glucose homeostases. Nature.

[49] Perry RJ (2014). Leptin reverses diabetes by suppression of the hypothalamic-pituitary-adrenal axis. Nat Med.

[50] Morton GJ, Meek TH, Matsen ME, Schwartz MW (2015). Evidence against hypothalamic-pituitary-adrenal axis suppression in the antidiabetic action of leptin. Journal of Clinical Investigation.

[51] Perry RJ (2017). Mechanism for leptin’s acute insulin-independent effect to reverse diabetic ketoacidosis. Journal of Clinical Investigation.

[52] Huang L, Wang Z, Li C (2001). Modulation of circulating leptin levels by its soluble receptor. J Biol Chem.

