# Supplementary material for: Metabolic effects of leptin receptor knockdown or reconstitution in adipose tissues
Source: Sci Rep. 2019 Mar 1;9:3307. doi: 10.1038/s41598-019-39498-3 (PMC6397253; doi:10.1038/s41598-019-39498-3)
Supplement: Supplementary file 1 — Supplementary Information [file 41598_2019_39498_MOESM1_ESM.pdf]

SUPPLEMENTARY INFORMATION

Metabolic effects of leptin receptor knockdown or reconstitution in adipose tissues

Sandra Pereira[1], Shannon M. O'Dwyer[1], Travis D. Webber[1], Robert K. Baker[1], Victor So[1], Cara E. Ellis[1], Ji Soo Yoon[1], Majid Mojibian[1], Maria M. Glavas[1], Subashini Karunakaran[1], Susanne M. Clee[1], Scott D. Covey[2], Timothy J. Kieffer[1,3*]

[1]Department of Cellular and Physiological Sciences, University of British Columbia, Vancouver, BC, Canada

[2]Department of Biochemistry and Molecular Biology, University of British Columbia, Vancouver, BC, Canada

[3]Department of Surgery, University of British Columbia, Vancouver, BC, Canada

SUPPLEMENTARY METHODS

Genotyping
Genotyping was performed using the following primers for endpoint PCR or primers and probes for qPCR:

AdipoqCre genotyping:
Endpoint PCR (*AdipoqCre*):
Forward primer (mAdipoq-F): GAGTATGTAGATGCAGATCTTTGGAGTG
Reverse primer (CreER-R1): CAAACGGACAGAAGCATTTTCCAG
qPCR (*AdipoqCre*):
Forward primer (qAdipoq-F2): GTC TGC CTT TCC CAT GAC TAT T
Reverse primer (qAdipoq-R1): TGG TGT ACG GTC AGT AAA TTG G
Probe (qAdipoq-P1): TGC CCA AGA AGA AGA GGA AGG TGT

Leprflox genotyping:
Endpoint PCR ($Lepr^{flox}$ and $Lepr^{+}$):
Forward primer (LepRF): ACACCACACTGTTGAGACACC
Reverse primer (LepRR): CATTTGATTCCACAAAGTGTTCCCTAAAC
qPCR ($Lepr^{flox}$):
Forward primer (Lepr Ex17-F): TTA ACC CAG ATA TCG AAT TCC T
Reverse primer (Lepr Ex17-R): GAG AAC ATG AAC ACA ACA ACA
Probe (Lepr Ex17-HEX): CCC GAT TTC GAA CCC GAC TCT AGA CT
qPCR ($Lepr^{+}$):
Forward primer (qLepr(WT)-F1): CTG GGT GAT CTC ACA CAT AC
Reverse primer (qLepr(WT)-R1): CAT AAG TCC ACG GGA TAT GG
Probe (qLepr(WT)-P1): AGT GAG GAG GGA AGA CGT TAT AAT CT

LeprloxTB genotyping:
Endpoint PCR ($Lepr^{loxTB}$):
Forward primer (15369): TGG CTT TTA AGC TCT GCA GTC
Reverse primer (15371): CCC AAG GCC ATA CAA GTG TT
Endpoint PCR ($Lepr^{+}$):
Forward primer (15369): TGG CTT TTA AGC TCT GCA GTC
Reverse primer (15370): TAG GGC CAA ACC CAC ATT TA
qPCR ($Lepr^{loxTB}$):
Forward primer (qLeprNull-F2): CTG CAT TCT AGT TGT GGT TTG
Reverse primer (qLeprNull-R2): GTC TCA TGA GCG GAT ACA TAT T
Probe (qLeprNull-P2): TCA TGT CTG GAT CGC TTA GGT GGC A
qPCR ($Lepr^{+}$):
Forward primer (qLepr(WT)-F2): GTG AGA TCA TGA GAC CCT AAA
Reverse primer (qLepr(WT)-R2): GGA ACT CAA GAC CAT CTA TCA
Probe (qLepr(WT)-P2): TTC TGA ATT GGT GTC CCT GGA GCC

mTmG genotyping:
Endpoint PCR (*mTmG*):
Forward primer (oIMR7318): CTC TGC TGC CTC CTG GCT TCT
Reverse primer (mTmG-MUT): ACC GTA AGT TAT GTA ACG CGG AAC TCC
Endpoint PCR (Wild-type):
Forward primer (oIMR7318): CTC TGC TGC CTC CTG GCT TCT
Reverse primer (mTmG-WT): CTC CGA GGC GGA TCA CAA GCA ATA
qPCR (*mTmG*:):
Forward primer (qmTmG-F1): CAT ATA TGG AGT TCC GCG TTA CA
Reverse primer (qmTmG-R1): GAA AGT CCC TAT TGG CGT TAC T
Probe (qmTmG-P1): TAA CTT ACG GTA AAT GGC CCG CCT
qPCR (*Wild-type (ROSA26 locus)*):
Forward primer (qROSA26-F2): TGA TCT GCA ACT CCA GTC TTT C
Reverse primer (qROSA26-R2): GGA AGT CTT GTC CCT CCA ATT T
Probe (qROSA26-P2): TTT AAG CCT GCC CAG AAG ACT CCC

Immunofluorescence analysis and hematoxylin and eosin (H&E) staining
Tissues from $AdipoqCre^{+}Lepr^{flox/flox}ROSA26^{mTmG/mTmG}$ and $AdipoqCre^{-}Lepr^{flox/flox}ROSA26^{mTmG/mTmG}$ mice that were 13-18 weeks old at time of euthanasia were fixed with 4% paraformaldehyde and embedded with paraffin for immunofluorescence analysis and H&E staining. Immunofluorescence analysis was performed as previously described [1] and the following antibodies were used: the primary antibody for green fluorescent protein (GFP) was a rabbit polyclonal antibody (1:500, Life Technologies, Carlsbad, CA, USA), while the secondary antibody was goat anti-rabbit AF488 (1:1000, Life Technologies).

Statistical analysis
For Fig. 6A-D, statistical analyses were performed in R version 3.4.2. A Gompertz function mixed effects model was fit to body weight using the function nlmer in package nlme [2], with random effects of individual mice contributing to the maximum body weight (asymptote) and fixed effects of Group predicting body weight at birth (x-intercept), asymptote, and sigmoidal growth rate. Starting values were generated using the getInitial function. A linear mixed effects model was fit to blood glucose using function lmer in package lme4 [3], with random effects of individual mice contributing to the intercept at each time point and time treated as a categorical variable. Pairwise comparisons were performed using the function emmeans or emtrends from the package emmeans [4] with the Kenward-Roger modification of the F-statistic [5], with corrections for multiple comparisons performed using Tukey's method. Models were diagnosed by examination of residual plots, and no serious deviations from normality were observed. Three values in the males were missing completely at random and were ignored as permitted by the mixed effects model approach. Figures were generated using the package ggplot2 [6]. Code used for these analyses is available upon request.

SUPPLEMENTARY RESULTS

Supplementary Figure S1. Raw blots, with bp of DNA ladder, of Figure 1 A-E: Endpoint PCR indicating the location on the gel of *Lepr*^{flox} (1369bp) and the allele resulting from Cre-induced excision, *Lepr*$^{\Delta 17}$ (952bp), in white adipose tissue (WAT), brown adipose tissue (BAT), adipocytes isolated from WAT, and non-adipose tissues in male and female *AdipoqCre*$^{+}$*Lepr*$^{flox/flox}$ (+) and *AdipoqCre*$^{-}$*Lepr*$^{flox/flox}$ (-) mice**.** For B, only a portion of the raw gel was used in Figure 1.

A: Male adipose tissues

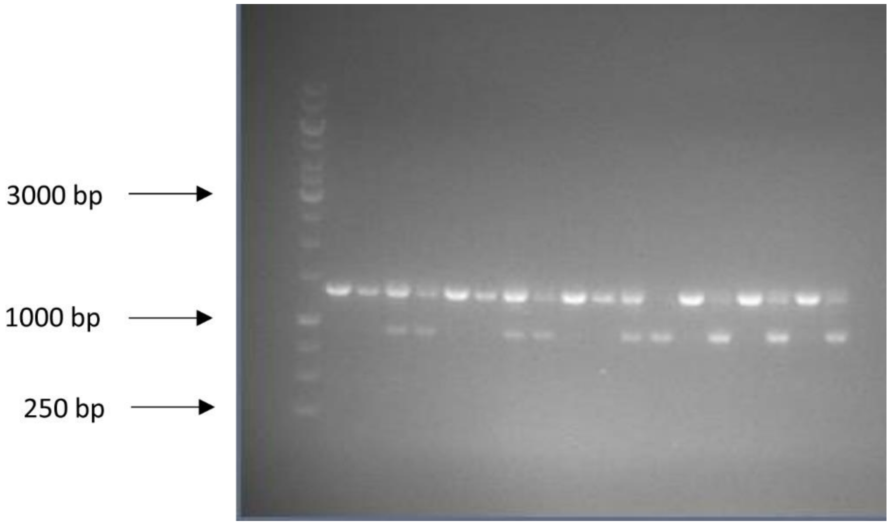

B: Female adipose tissues

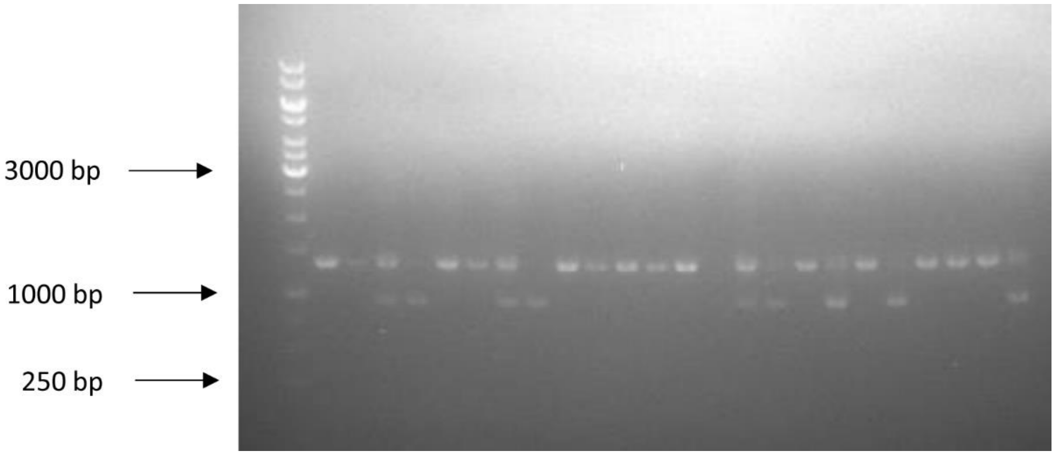

C: Adipocytes from WAT of males and females

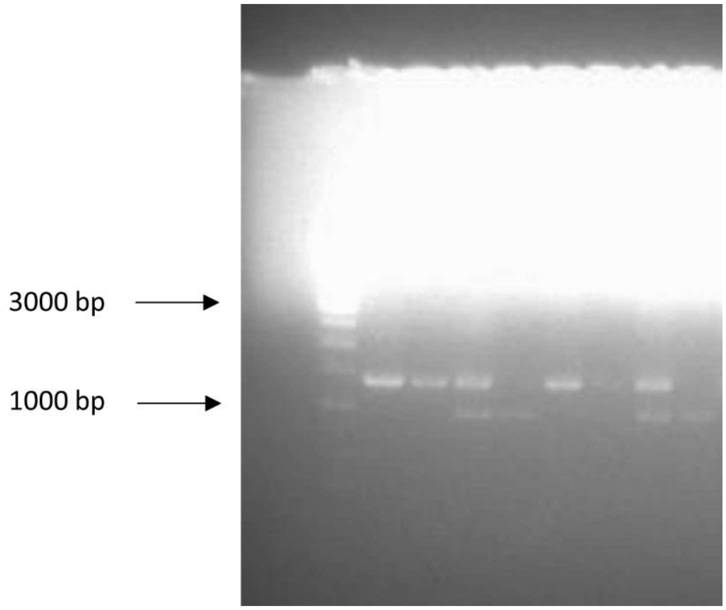

D and E: Male and Female tissues

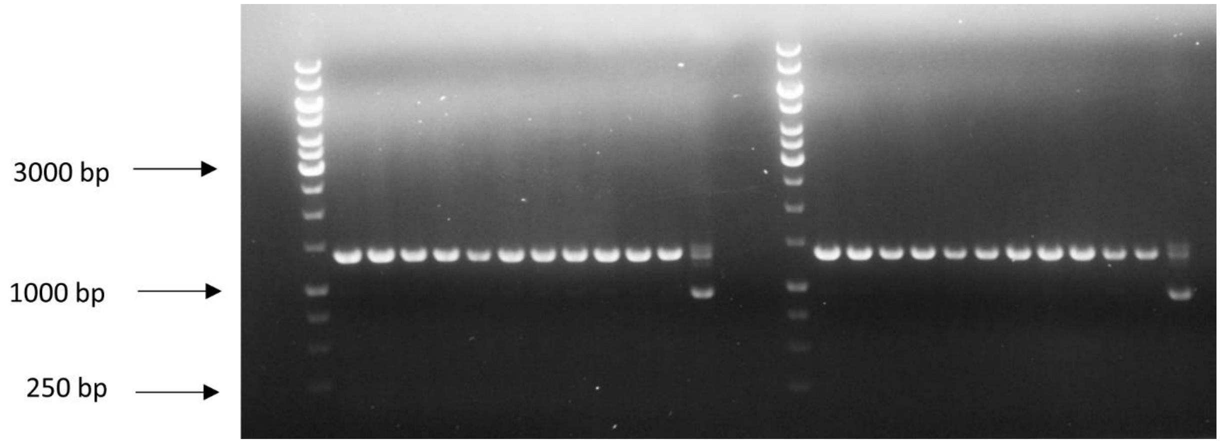

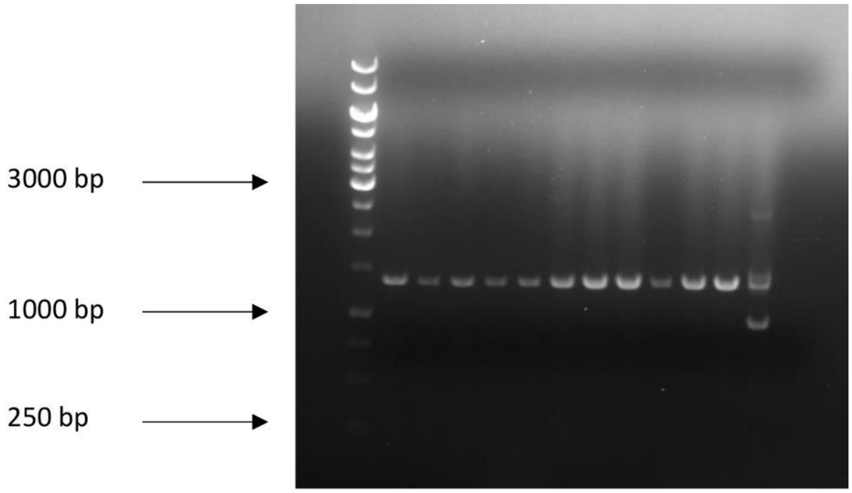
3000 bp
1000 bp
250 bp

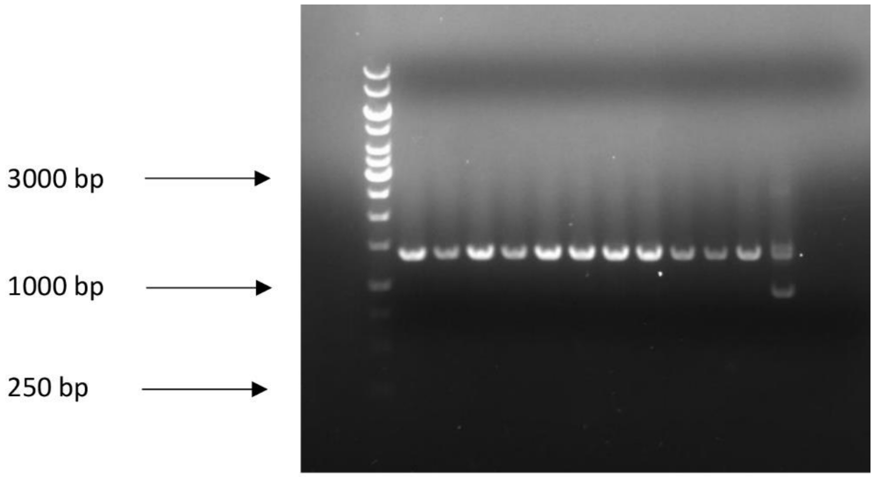
3000 bp
1000 bp
250 bp

Supplementary Figure S2. Endpoint PCR of *Lepr*^{flox} (1369bp) and the allele resulting from Cre-induced excision, *Lepr*$^{\Delta 17}$ (952bp), of duodenum mucosa (D), jejunum mucosa (J), ileum mucosa (I), and brown adipose tissue (BAT) from female *AdipoqCre*$^{+}$*Lepr*$^{flox/flox}$ (+) and *AdipoqCre*$^{-}$ *Lepr*$^{flox/flox}$ (-) mice**.** One (-) sample and one (+) sample from each tissue type was also run in gel of Figure 1E.

A: Tissues from *AdipoqCre*$^{+}$*Lepr*$^{flox/flox}$ and *AdipoqCre*$^{-}$*Lepr*$^{flox/flox}$ mice

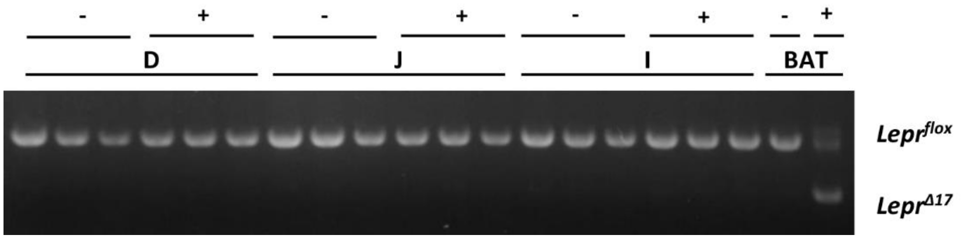

B: Raw blot of Supplementary Figure S2A

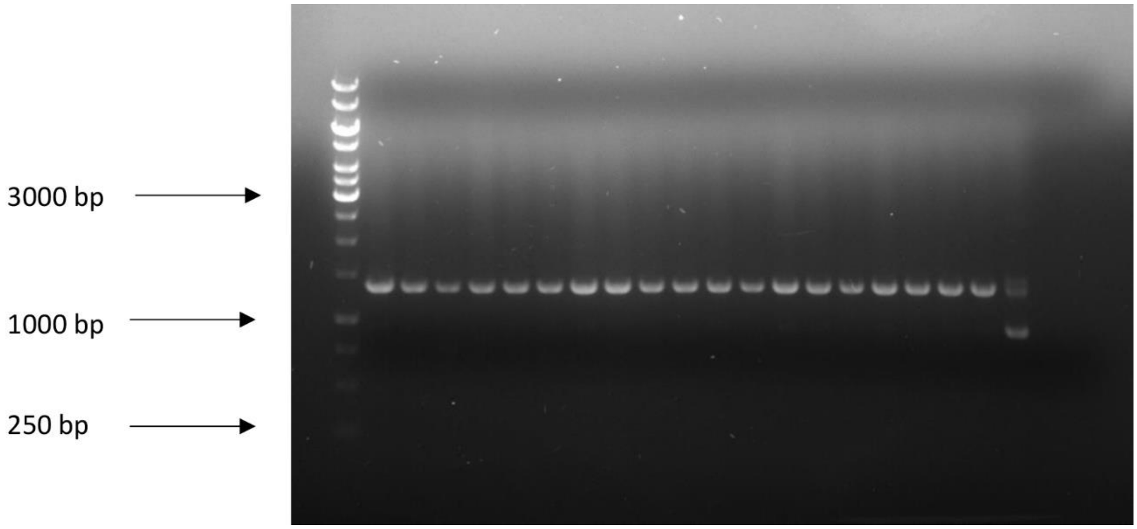

Supplementary Figure S3. A-D: Green fluorescent protein (GFP, green) immunofluorescence and 4’,6-diamidino-2-phenylindole (DAPI, white) in white adipose tissue (WAT) of male and female $AdipoqCre^{+}Lepr^{flox/flox}ROSA26^{mTmG/mTmG}$ ($AdipoqCre^{+}Lepr^{flox/flox}mTmG$) and $AdipoqCre^{-}Lepr^{flox/flox}ROSA26^{mTmG/mTmG}$ ($AdipoqCre^{-}Lepr^{flox/flox}mTmG$) mice. Scale bar is 100 µm. Enlarged section also shown. **E-J**: H&E staining (10X magnification) of WAT and brown adipose tissue (BAT) of male and female $AdipoqCre^{+}Lepr^{flox/flox}ROSA26^{mTmG/mTmG}$ and $AdipoqCre^{-}Lepr^{flox/flox}ROSA26^{mTmG/mTmG}$ mice. pg, perigonadal; sc, subcutaneous.

A Males, pgWAT

$AdipoqCre^{+}Lepr^{flox/flox}mTmG$ *$AdipoqCre^{-}Lepr^{flox/flox}mTmG$*

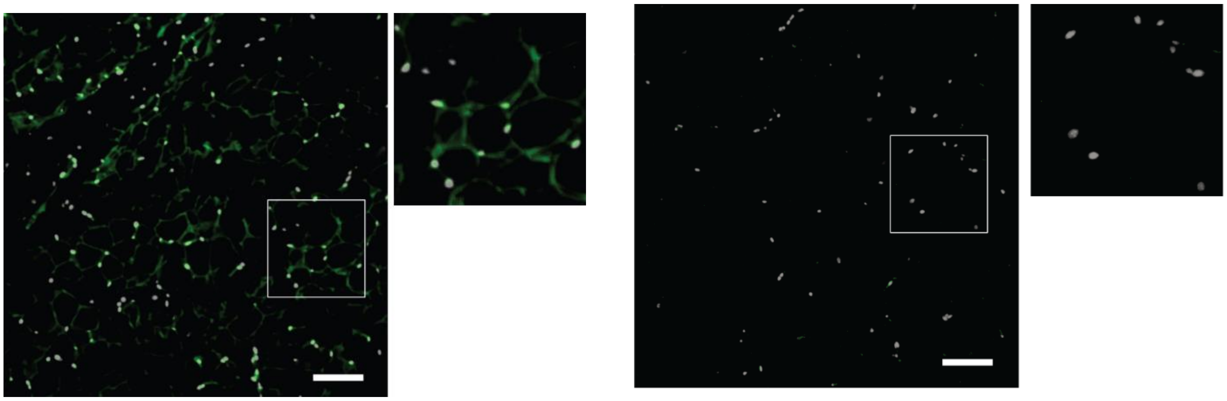

B Males, scWAT

$AdipoqCre^{+}Lepr^{flox/flox}mTmG$ *$AdipoqCre^{-}Lepr^{flox/flox}mTmG$*

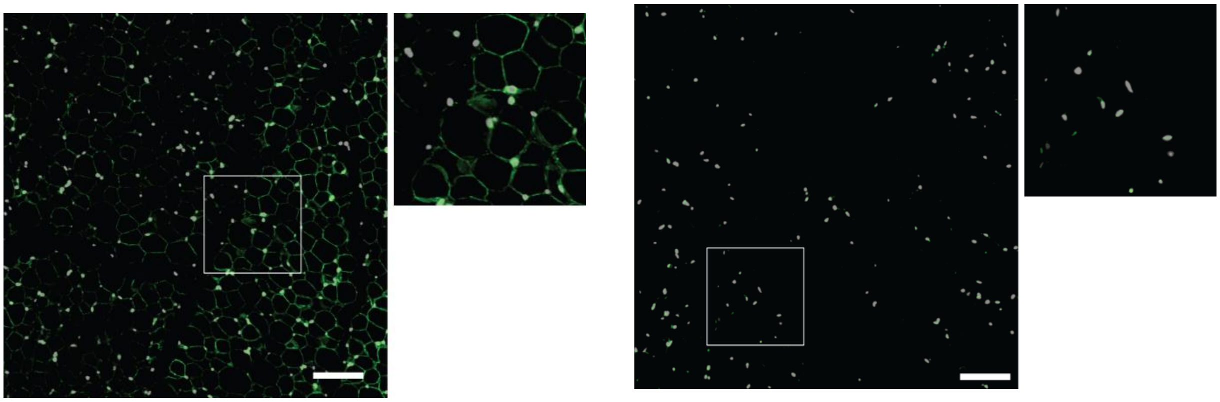

C Females, pgWAT

$\mathit{AdipoqCre}^{+}\mathit{Lepr}^{flox/flox}\mathit{mTmG}$

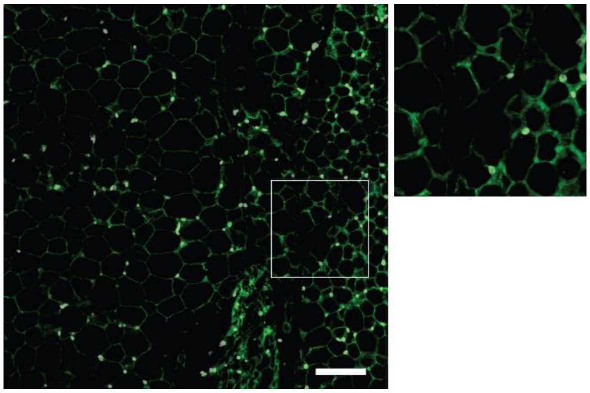

$\mathit{AdipoqCre}^{-}\mathit{Lepr}^{flox/flox}\mathit{mTmG}$

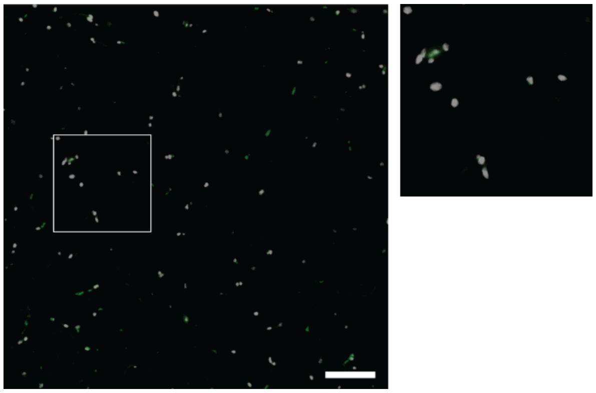

D Females, scWAT

$\mathit{AdipoqCre}^{+}\mathit{Lepr}^{flox/flox}\mathit{mTmG}$

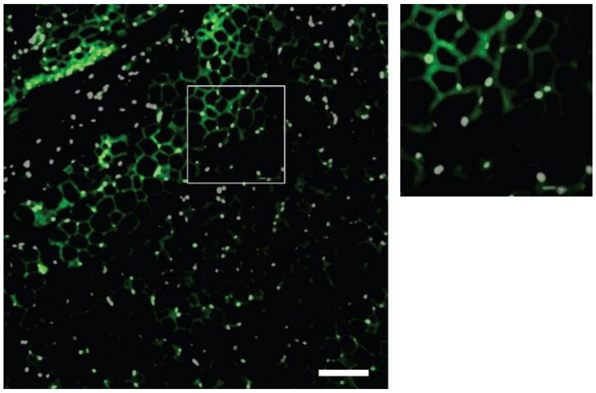

$\mathit{AdipoqCre}^{-}\mathit{Lepr}^{flox/flox}\mathit{mTmG}$

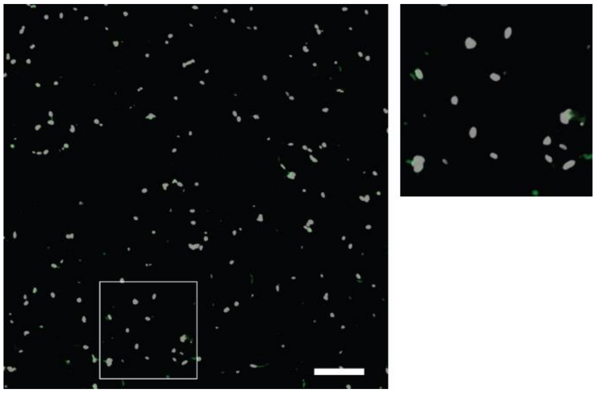

E Males, pgWAT

AdipoqCre^{+}*Lepr*$^{flox/flox}$*mTmG*

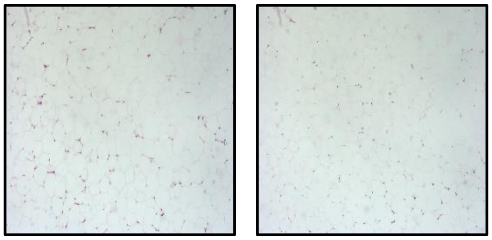

AdipoqCre^{-}*Lepr*$^{flox/flox}$*mTmG*

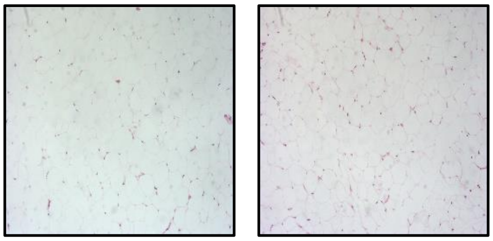

F Males, scWAT

AdipoqCre^{+}*Lepr*$^{flox/flox}$*mTmG*

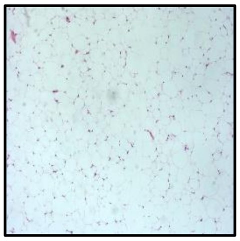

AdipoqCre^{-}*Lepr*$^{flox/flox}$*mTmG*

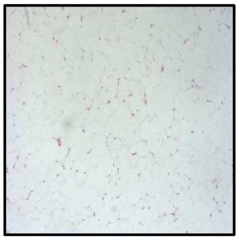

G Males, BAT

AdipoqCre^{+}*Lepr*$^{flox/flox}$*mTmG*

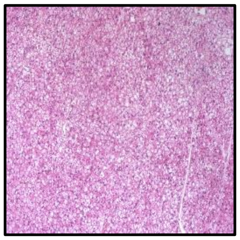

AdipoqCre^{-}*Lepr*$^{flox/flox}$*mTmG*

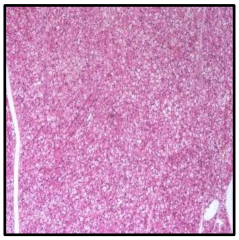

H Females, pgWAT

AdipoqCre^{+}*Lepr*$^{flox/flox}$*mTmG*

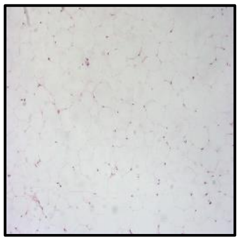

AdipoqCre^{-}*Lepr*$^{flox/flox}$*mTmG*

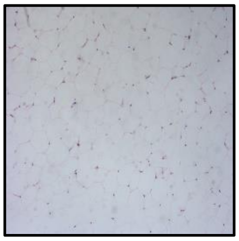

I Females, scWAT

AdipoqCre^{+}*Lepr*$^{flox/flox}$*mTmG*

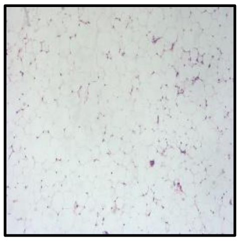

AdipoqCre^{-}*Lepr*$^{flox/flox}$*mTmG*

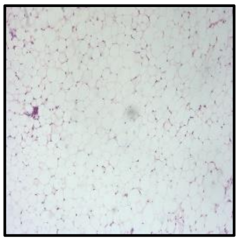

J Females, BAT

AdipoqCre^{+}*Lepr*$^{flox/flox}$*mTmG*

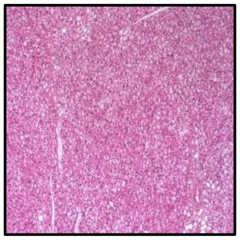

AdipoqCre^{-}*Lepr*$^{flox/flox}$*mTmG*

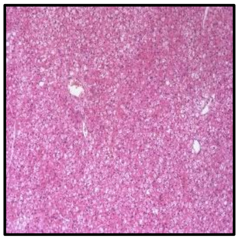

Supplementary Figure S4. Full Western blots, with protein ladder (Fisher scientific, catalogue# BP3603500), of results in Figure 5 A. Liver samples from mice injected with insulin or vehicle via the portal vein were prepared for Western blot analysis and probed for phospho-Akt (Ser473) (**A**) and total Akt (**B**). Membranes were probed for phospho-Akt, stripped, and then reprobed for total Akt. Gels were 10% polyacrylamide and expected molecular weight for phospho-Akt and total Akt is 60 kDa. Representative blots for phospho-Akt and total Akt in Figure 5 A correspond to the first 4 lanes of samples in each Membrane #1. In Membrane #1, samples were loaded in pairs based on treatment group, starting with vehicle-injected closest to protein ladder, then insulin-injected, followed by vehicle-injected, and lastly, insulin-injected. In Membrane #2, the 2 samples closest to the protein ladder are from vehicle-injected mice, while the rest are from insulin-injected mice. In all membranes, the loading order, starting with the sample closest to the protein ladder, is always *AdipoqCre^{-}Lepr$^{flox/flox}$* (-) and then *AdipoqCre^{+}Lepr$^{flox/flox}$* (+) mice.

A: Western blots probed for phospho-Akt (Ser473)

Membrane #1

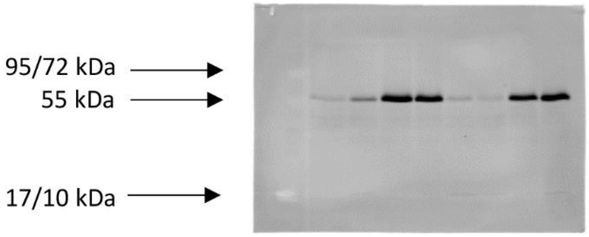

Membrane #2

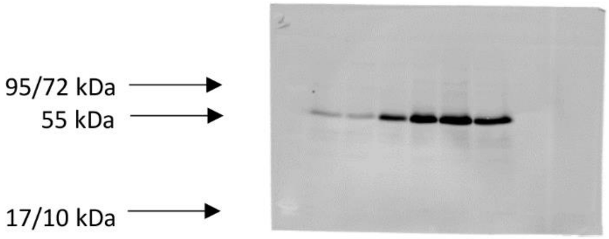

B: Western blots probed for total Akt

Membrane #1

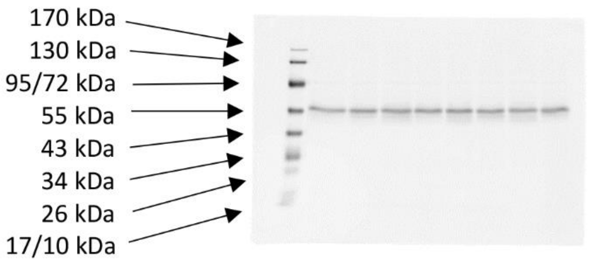

Membrane #2

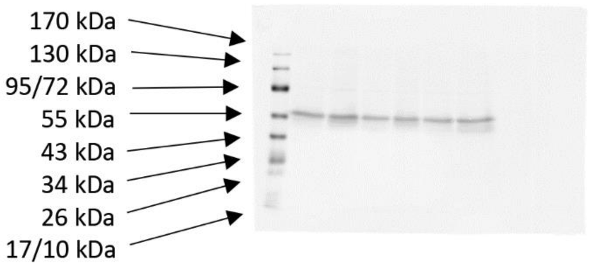

Supplementary Figure S5. Leptin secretion from perigonadal and subcutaneous white adipose tissue (pgWAT and scWAT, respectively) samples of *AdipoqCre⁻Lepr*$^{flox/flox}$ and *AdipoqCre*$^{+}$*Lepr*$^{flox/flox}$ male (**A**) and female (**B**) mice. Results refer to amount of leptin secreted into 200 µl of media over 2h at 37°C and are expressed per weight of tissue sample. In **A**, n=6 for *AdipoqCre*$^{-}$*Lepr*$^{flox/flox}$ and n=7 for *AdipoqCre*$^{+}$*Lepr*$^{flox/flox}$. In **B**, n=8 for *AdipoqCre*$^{-}$*Lepr*$^{flox/flox}$ and n=9 for *AdipoqCre*$^{+}$*Lepr*$^{flox/flox}$. Unpaired t-tests were performed for leptin secreted by each tissue type, for each sex.

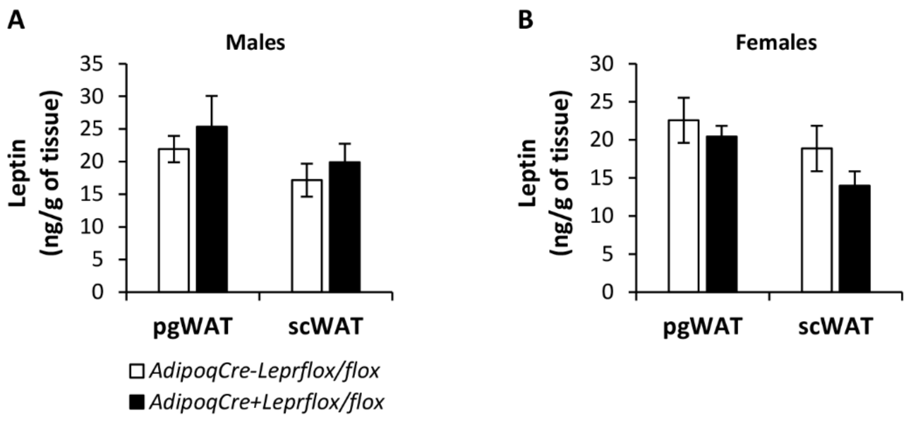

Supplementary Figure S6. Blood glucose (**A**) and body weight (**B**) in STZ-diabetic male mice treated with leptin or vehicle delivered by subcutaneous mini-osmotic pumps for 8 days. Plasma leptin concentrations (**C**), plasma leptin receptor concentrations on Day 8 (**D**), and plasma insulin concentrations on Day 8 (**E**). Plasma obtained from saphenous vein blood on Day -1 and cardiac blood on Day 8. Samples sizes: *AdipoqCre*$^{-}$*Lepr*$^{flox/flox}$, Sham (n=6, except for leptin receptor, where n=5); *AdipoqCre*$^{-}$*Lepr*$^{flox/flox}$, STZ+vehicle (n=6, except for leptin receptor and insulin, where n=5); *AdipoqCre*$^{-}$*Lepr*$^{flox/flox}$, STZ+leptin (n=10, except for leptin receptor, where n=9, and insulin, where n=5); *AdipoqCre*$^{+}$*Lepr*$^{flox/flox}$, Sham (n=3); *AdipoqCre*$^{+}$*Lepr*$^{flox/flox}$, STZ+vehicle (n=5, except for leptin receptor and insulin, where n=4); *AdipoqCre*$^{+}$*Lepr*$^{flox/flox}$, STZ+leptin (n=9, except for insulin, where n=4). Some insulin levels were too low to be detected by the insulin ELISA, as specified above. Repeated measures two-way ANOVA (**A** and **B**) or one-way ANOVA (**C**, **D**, and **E**) were performed, as described in the Methods section. *p<0.05, Sham controls vs. other treatments. †p<0.05, *AdipoqCre*$^{-}$*Lepr*$^{flox/flox}$ STZ+vehicle vs. *AdipoqCre*$^{+}$*Lepr*$^{flox/flox}$ STZ+leptin. ‡p<0.05, *AdipoqCre*$^{-}$*Lepr*$^{flox/flox}$ STZ+leptin vs. *AdipoqCre*$^{+}$*Lepr*$^{flox/flox}$ STZ+leptin. §p<0.05, *AdipoqCre*$^{+}$*Lepr*$^{flox/flox}$ STZ+vehicle vs. *AdipoqCre*$^{+}$*Lepr*$^{flox/flox}$ STZ+leptin. ‖p<0.05, *AdipoqCre*$^{-}$*Lepr*$^{flox/flox}$ STZ+vehicle vs. *AdipoqCre*$^{-}$*Lepr*$^{flox/flox}$ STZ+leptin. #p<0.05, STZ+vehicle groups vs. STZ+leptin groups. **p<0.05, Sham vs. all groups, except other Sham and *AdipoqCre*$^{+}$*Lepr*$^{flox/flox}$ STZ+leptin. ††p<0.05, *AdipoqCre*$^{-}$*Lepr*$^{flox/flox}$ Sham vs. *AdipoqCre*$^{-}$*Lepr*$^{flox/flox}$ STZ+leptin. ‡‡p<0.05, *AdipoqCre*$^{-}$*Lepr*$^{flox/flox}$ Sham vs. STZ+leptin groups. §§, p<0.05, *AdipoqCre*$^{+}$*Lepr*$^{flox/flox}$ Sham vs. *AdipoqCre*$^{-}$*Lepr*$^{flox/flox}$ STZ+leptin. ‖‖p<0.05, *AdipoqCre*$^{+}$*Lepr*$^{flox/flox}$ Sham vs. *AdipoqCre*$^{+}$*Lepr*$^{flox/flox}$ STZ+leptin. ¶p<0.05 vs. all Shams. ##p<0.05 vs *AdipoqCre*$^{+}$*Lepr*$^{flox/flox}$ Sham. ***p<0.05 vs. all other groups.

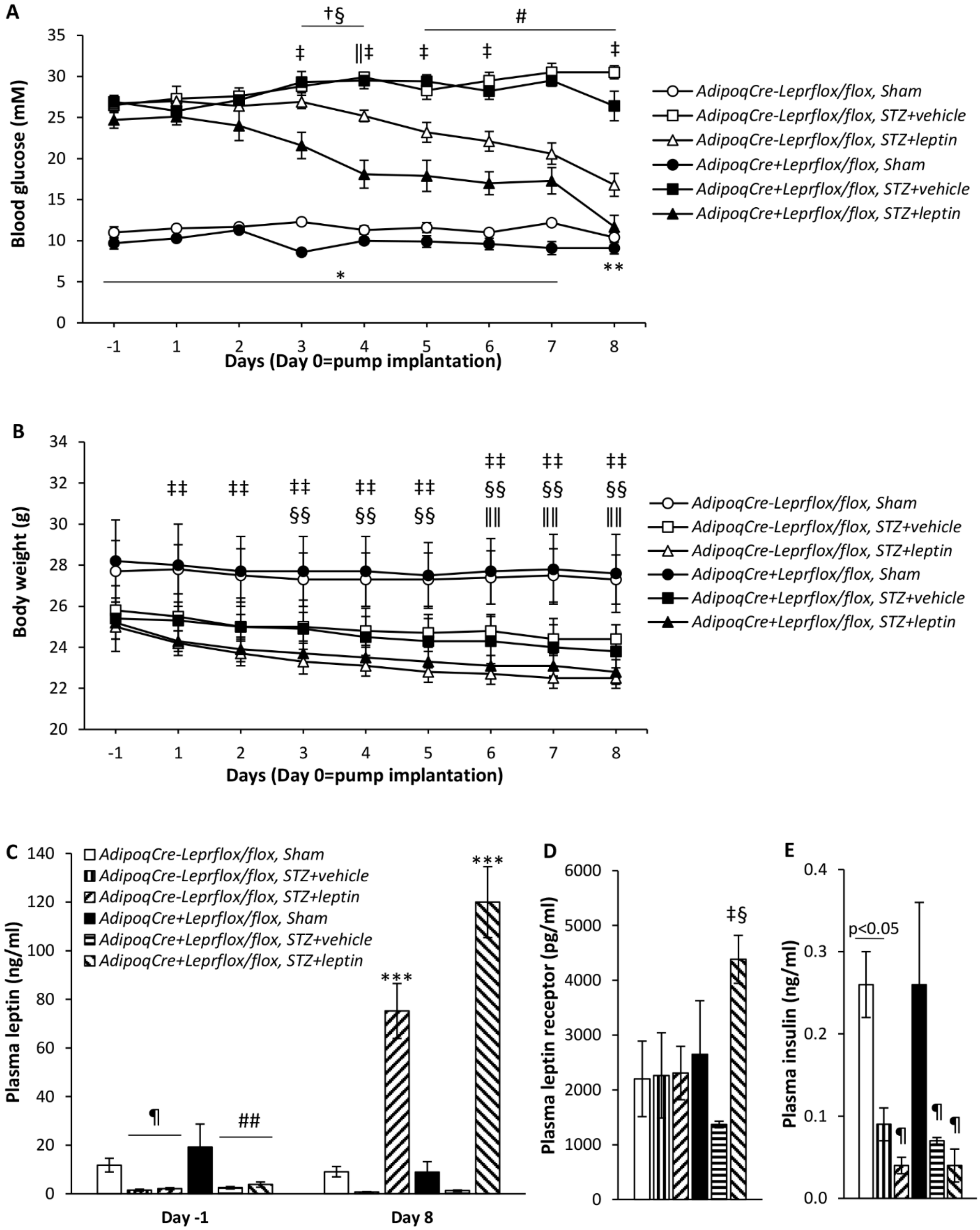
A
B
C
D
E
Blood glucose (mM)
Days (Day 0=pump implantation)
Body weight (g)
Plasma leptin (ng/ml)
Plasma leptin receptor (pg/ml)
Plasma insulin (ng/ml)
AdipoqCre-Leprflox/flox, Sham
AdipoqCre-Leprflox/flox, STZ+vehicle
AdipoqCre-Leprflox/flox, STZ+leptin
AdipoqCre+Leprflox/flox, Sham
AdipoqCre+Leprflox/flox, STZ+vehicle
AdipoqCre+Leprflox/flox, STZ+leptin
Day -1
Day 8
p<0.05

Supplementary Figure S7. Blood glucose (**A**) and plasma leptin (**B**) in STZ-diabetic *AdipoqCre⁻Lepr*$^{flox/flox}$ and *AdipoqCre*$^{+}$*Lepr*$^{flox/flox}$ male mice treated with a single injection of leptin. Results were obtained immediately before leptin injection (0h) and at multiple times following injection. n=5 per genotype. A repeated measures two-way ANOVA with Bonferroni *post-hoc* test was carried out in **A** and in **B**. In **A** and **B**, there is a main effect of time ($p<0.05$).

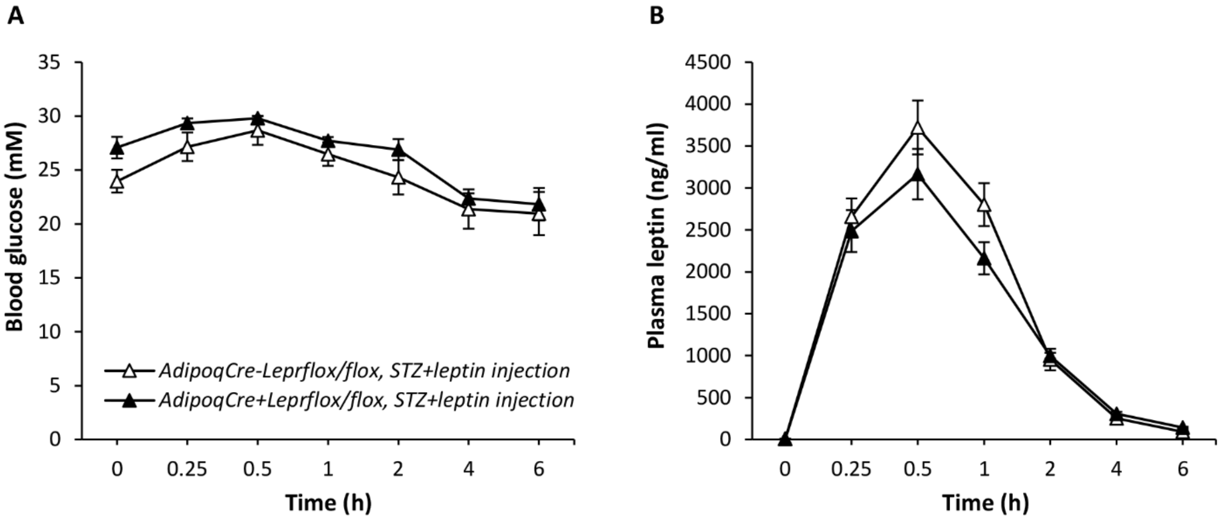

Supplementary Figure S8. Estimates of body weight at birth (x-intercept), maximum body weight (asymptote), and growth rate in males (**A**) and females (**B**) from the ATLeprEXP colony, obtained using statistical analysis in R. Values in brackets are 95% confidence intervals.

A Males

Genotype	x-intercept (g)	Asymptote (g)	Growth rate
AdipoqCre^{-}*Lepr*$^{loxTB/TB}$	2.1 (1.9, 2.3)	64.4 (61.6, 67.2)	0.82 (0.80, 0.84)
AdipoqCre^{+}*Lepr*$^{loxTB/TB}$	2.0 (1.9, 2.2)	71.3 (68.2, 74.4)	0.83 (0.82, 0.85)
AdipoqCre^{+}*Lepr*$^{+/+}$	1.2 (1.1, 1.2)	42.1 (35.6, 48.7)	0.91 (0.88, 0.94)
AdipoqCre^{-}*Lepr*$^{+/+}$	1.2 (1.1, 1.3)	43.5 (37.3, 49.6)	0.90 (0.86, 0.93)

B Females

Genotype	x-intercept (g)	Asymptote (g)	Growth rate
AdipoqCre^{-}*Lepr*$^{loxTB/TB}$	2.1 (2.0, 2.3)	72.1 (69.3, 74.8)	0.84 (0.82, 0.85)
AdipoqCre^{+}*Lepr*$^{loxTB/TB}$	2.2 (1.9, 2.4)	71.9 (68.2, 75.6)	0.83 (0.82, 0.85)
AdipoqCre^{+}*Lepr*$^{+/+}$	1.1 (0.9, 1.2)	30.7 (24.3, 37.2)	0.89 (0.83, 0.95)
AdipoqCre^{-}*Lepr*$^{+/+}$	1.1 (1.0, 1.2)	33.8 (24.0, 43.6)	0.91 (0.85, 0.97)

Supplementary Figure S9. Oral glucose tolerance test (OGTT, 1g/kg) at 23-27 weeks of age (**A**) and i.p. glucose tolerance test (IPGTT, 1g/kg) at 27-29 weeks of age (**B**) in male *AdipoqCre⁻ Lepr*$^{loxTB/loxTB}$ and *AdipoqCre⁺Lepr*$^{loxTB/loxTB}$ mice. For each test, blood glucose concentration is on the left and plasma insulin concentration is on the right. n=5 for *AdipoqCre⁻Lepr*$^{loxTB/loxTB}$ and n=3 for *AdipoqCre⁺Lepr*$^{loxTB/loxTB}$ mice. For each parameter measured over time, repeated measures two-way ANOVA with Bonferroni *post-hoc* test was carried out. For each comparison of area under the curve (AUC), an unpaired t-test was performed, except for blood glucose and plasma insulin in **A**, where the Mann-Whitney ***U*** test was performed. In **A** and **B**, for blood glucose and plasma insulin, there is a main effect of time ($p<0.05$). *$p<0.05$, *AdipoqCre⁺Lepr*$^{loxTB/loxTB}$ vs *AdipoqCre⁻Lepr*$^{loxTB/loxTB}$.

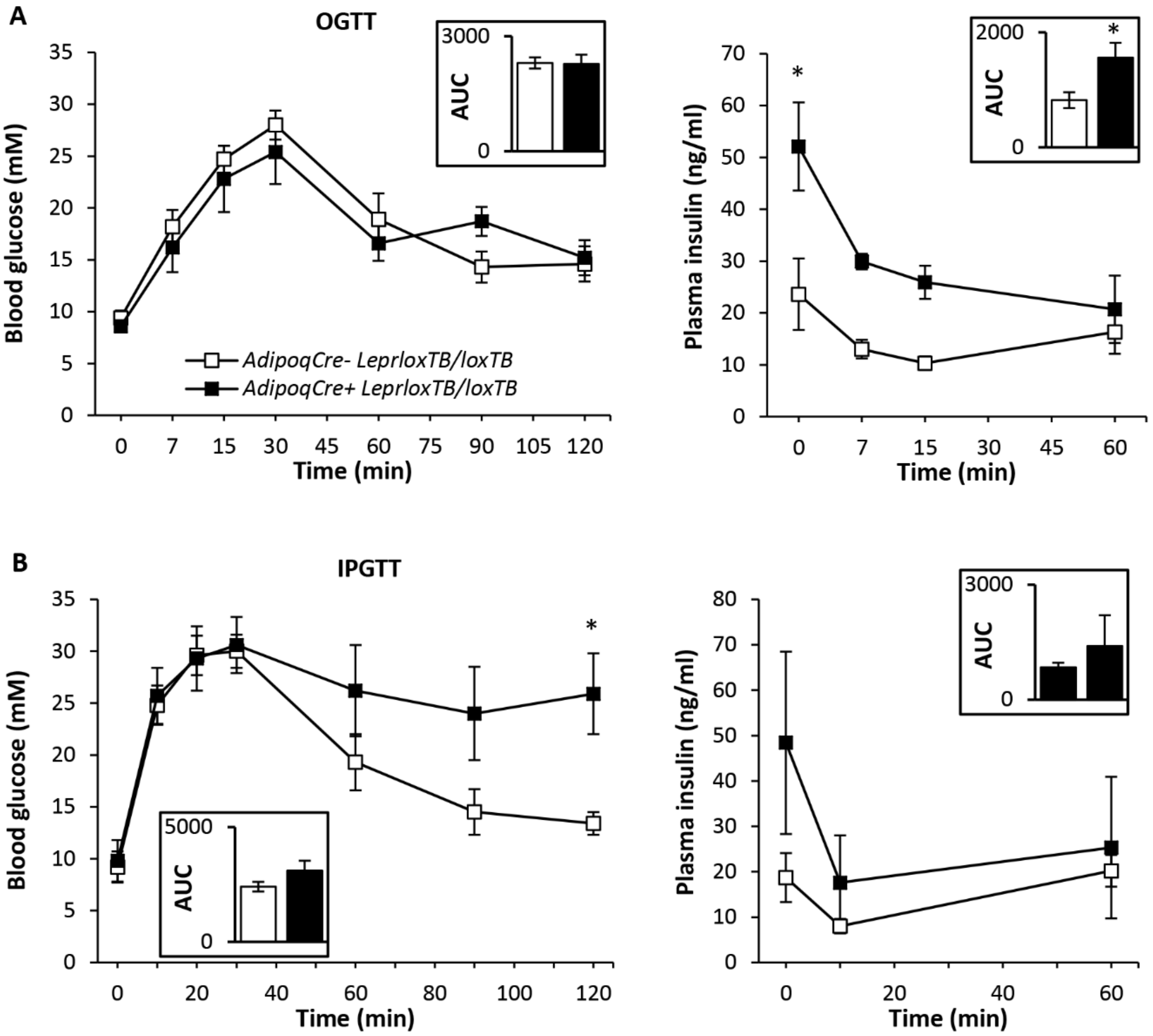

SUPPLEMENTAL REFERENCES

1. Tuduri, E., *et al.* Partial ablation of leptin signaling in mouse pancreatic alpha-cells does not alter either glucose or lipid homeostasis. *American Journal of Physiology - Endocrinology & Metabolism* **306**, E748-755 (2014).
2. Pinheiro, J., Bates, D., DebRoy, S., Sarkar, D. & Team, R.C. _nlme: Linear and Nonlinear Mixed Effects Models_. R package version 3.1-131. (2017).
3. Bates, D., Maechler, M., Bolker, B. & Walker, S. Fitting linear mixed-effects models using lme4. *Journal of Statistical Software* **67**, 1-48 (2015).
4. Lenth, R. emmeans: Estimated marginal means, aka least-squares means. R package version 0.9.1. (2017).
5. Kenward, M.G. & Roger, J.H. Small sample inference for fixed effects from restricted maximum likelihood. *Biometrics* **53**, 983-997 (1997).
6. Wickham, H. *ggplot2: Elegant graphics for data analysis*, (Springer-Verlag New York, 2009).
